# Lactate in Anaphylaxis: 100 Years On

**DOI:** 10.1007/s40279-025-02273-8

**Published:** 2025-07-24

**Authors:** Elena Borzova, Bernhard F. Gibbs

**Affiliations:** 1 Dermatology Division, Niigata University Graduate School of Medical and Dental Sciences, 1-757 Asahimachi-dori, Chuo-ku, Niigata, 951-8510 Japan; 2 School of Psychology and Life Sciences, Canterbury Christ Church University, North Holmes Road, Canterbury, Kent CT1 1QU UK

## Abstract

Lactate is a ubiquitous and pleiotropic signalling molecule, with important functional effects in tissue and cellular metabolism. As an exerkine, lactate is not only substantially released from tissues during exercise but may also play a role in exercise-related medical conditions. Since exercise is a known co-factor in anaphylaxis, this may suggest a mechanistic relevance for lactate release during anaphylactic events. Here, we evaluated in vitro and in vivo evidence for lactate release from mast cells (MCs) from preclinical microdialysis studies, animal models and clinical data in anaphylaxis. Lactate levels are markedly elevated in both animal models of anaphylaxis and patients with anaphylaxis. Although not causative, lactate is clearly relevant to many different steps in anaphylaxis, including MC activation, vascular permeability and gastrointestinal dysfunction, but currently lacks a comprehensive interpretation framework in anaphylaxis. As a result, lactate cannot be currently considered a biomarker of anaphylaxis per se. However, given its wide dynamic range, a plethora of available lactate biosensors, and the ease of measurement in various biological fluids, lactate may be a potential candidate for biomarker development. At present, lactate’s contribution to anaphylaxis, which was discovered a century ago by Zunz and La Barre, has not been fully elucidated. Unresolved issues in anaphylaxis include the patient’s metabolic state; the kinetics of lactate release and its biological actions; MC bioenergetics and metabolome; activation thresholds; and feedback mechanisms, as well as an expression of lactate-metabolizing enzymes. A closer focus on these known unknowns may demystify the contribution of lactate in anaphylaxis and beyond.

**Graphical Abstract:**

Lactate in anaphylaxis. There are various clinical scenarios for lactate measurements in anaphylaxis: (1) exercise-induced anaphylaxis, (2) fatal or near-fatal anaphylaxis, (3) anaphylaxis in mastocytosis and (4) epinephrine-induced lactic acidosis. First, there is likely to be an overlap in early signalling events and pre- and post-translational processes mediated by lactate in the context of exercise in healthy subjects and in patients with exercise-induced urticaria/anaphylaxis, which may well be further complicated by abnormal MC reactivity, activation threshold and, possibly, feedback mechanisms in the latter. This underlines a commonality in metabolic pathways that may involve, at least in part, MC-derived histamine and its subsequent hemodynamic effects involved in anaphylaxis. Second, elevated serum lactate was demonstrated to be associated with anaphylaxis severity [54], especially in relation to fatal anaphylaxis in critical care settings [31]. This is in keeping with circumstantial evidence from a variety of previous reports from case studies and animal models. Furthermore, lactate release parallels histamine release in a MC activation event in a patient with mastocytosis [147]. Finally, lactate acidosis may rarely occur following multiple epinephrine injections in patients with anaphylaxis.

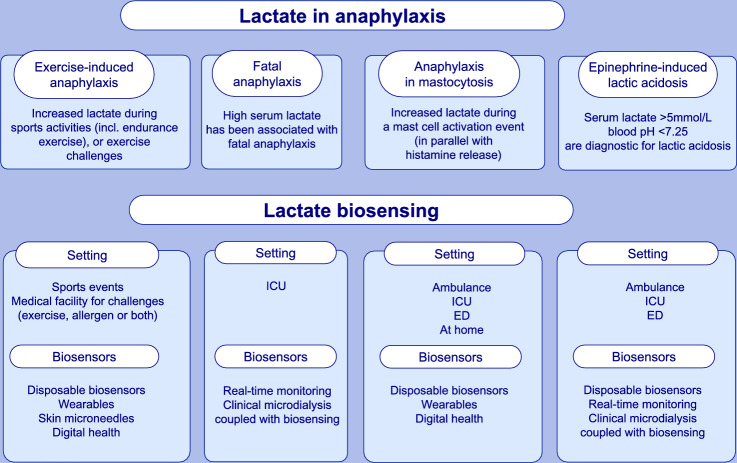

**Supplementary Information:**

The online version contains supplementary material available at 10.1007/s40279-025-02273-8.

## Key Points

**Table:** 

Lactate, albeit not causal, may be associated with the severity of anaphylactic reactions due to upregulated glycolytic pathways.
Glycolytic pathways play a pivotal role in endothelial hyperpermeability and are triggered by histamine, which is released both physiologically during exercise as well as by allergic mechanisms.
There is a need for further metabolomic studies in human and murine anaphylaxis.

## Introduction

### Anaphylaxis: Spotlight on the Unmet Needs

Anaphylaxis is the most severe sudden-onset systemic allergic reaction which is unpredictable in severity [1] and is potentially life-threatening [2]. Owing to a spectrum of clinical manifestations with multisystem organ involvement [3], anaphylaxis has various definitions [4]. The annual incidence of anaphylaxis has substantially increased worldwide over the past decades [5, 6], raising concerns over an anaphylaxis ‘epidemic’ [7]. Emergency department visits and hospitalizations due to anaphylaxis have been increasing in the UK, the USA [8] and Australia [9].

The mechanisms of anaphylaxis are primarily caused by allergen-mediated crosslinking of immunoglobulin E (IgE) bound to high-affinity IgE receptors (FcεRI) on mast cells and basophils and the resultant release of histamine as well as de novo-generated inflammatory lipid mediators. Histamine and platelet-activating factor (PAF) majorly contribute to vasodilatation, bronchoconstriction and atrial fibrillation, all of which potentially contribute to the severity of systemic anaphylaxis [10–12]. In addition, cysteinyl leukotrienes are also rapidly produced from mast cells and basophils, where they contribute to bronchoconstriction, and have been clearly implicated in anaphylaxis [13].

Although relatively rare, fatalities due to anaphylaxis may occur because of shock, laryngeal angioedema with asphyxia, severe bronchospasm and cardiac arrythmias [14]. Data from the European Anaphylaxis Registry suggests that the rate of fatal drug anaphylaxis has been increasing [15]. Although fatal outcomes are rare, adolescents and young adults are particularly vulnerable to fatal food-induced anaphylaxis [16], whereas fatal anaphylaxis occurs in older adults, with a median age of 49 years [15].

Exercise-induced hypersensitivity disorders, including exercise-induced anaphylaxis (EIA) (Box 1), are also recognized as an important problem in recreational and competitive athletes [17]. Cold-induced anaphylaxis occurs in 37% of patients with typical cold urticaria according to the international COLD-CE study [18]. Anaphylaxis is thus an area of unmet clinical need, requiring prompt recognition, immediate management and more effective preventive strategies.

**Box 1:** Exercise and anaphylaxis

The relationship between exercise and anaphylaxis can be divided into three phenotypes:
**1. Classical anaphylaxis** At least in some individuals, it can be affected by exercise **2. Food-dependent exercise-induced anaphylaxis** Food-induced reactions occur in the context of exercise. Typically, food ingestion is followed by exercise **3. Exercise-induced anaphylaxis** Exercise is thought to trigger the anaphylactic episode, independent of an exogenous allergen

### Anaphylaxis: Why Do We Need Biomarkers?

Biomarker development in anaphylaxis is intended to aid with the retrospective diagnosis in patients with ambiguous presentations or in cases of idiopathic anaphylaxis. In allergy practice, some patients with a history of anaphylaxis may present with a paucity of the data regarding the index episode [19]. Importantly, there is a need for risk stratification to identify the patients at risk of severe anaphylaxis [20]. In post-mortem examinations of patients with sudden death, biomarkers could help identify the cases with fatal anaphylaxis [19]. Perioperative anaphylaxis needs to be confirmed and followed up with a detailed allergy work-up for suspected drug allergies. There is also a need for prediction and early diagnosis of prolonged, refractory or biphasic anaphylaxis [20]. EIA needs to be differentiated from exercise-induced hypotension in collapsed endurance athletes, highlighting the need for reliable biomarkers in various clinical contexts where exercise can play a role in anaphylaxis [21, 22].

Multiple biomarkers have been in development to address these needs, but their performance in anaphylaxis depends on multiple factors, including testing systems, clinical settings, sampling time and causative allergens [19]. Tryptase is a useful biomarker of anaphylaxis, with a plasma half-life of approximately 2 h, which may remain elevated within 24–48 h of its onset, characterized by high specificity but variable sensitivity, depending on the trigger [23]. Individuals with hereditary α-tryptasemia, with increased α-tryptase-encoding *TPSAB1* copy number, may be at increased risk of severe anaphylaxis [24, 25], especially in patients with systemic mastocytosis [25, 26].

Urine biomarkers, including *N*-methyl histamine, prostaglandins (PGD2 and its metabolite PGF2α) and leukotrienes (LTE4) [27] are subject to wide inter-individual variability. Other biomarkers, such as chymase, carboxypeptidase A, platelet-activating factor, dipeptidyl peptidase I, basogranulin and CCL2, are currently in development [19]. Skin tests are important diagnostic tools which are usually carried out within 6 months of an anaphylaxis episode [28] but, as with assessing allergen-specific IgE, they do not serve as biomarkers for anaphylaxis. Cytokines such as TNF-α, IL-6 and IL-1β are also thought to play a role in anaphylaxis [23]. However, existing biomarkers of anaphylaxis have known limitations [29] and are either not commercially available or not available at the point-of-care [30]. The search for biomarkers of anaphylaxis is an ongoing issue [7], highlighting an important knowledge gap in anaphylaxis research [20]. Recent work from France suggested an association between lactate and fatal anaphylaxis in critical care settings [31], suggesting its potential utility as a biomarker to guide the management of severe anaphylaxis.

### Lactate and Anaphylaxis: A Potential Biomarker?

Lactate is a ubiquitous messenger, regulating metabolism and physiology [32]. It is produced from the glycolytic intermediate, pyruvate, due to the actions of lactate dehydrogenase (LDH). Under aerobic conditions, pyruvate is usually converted to acetyl CoA and enters the Kreb’s cycle to produce NADH and FADH2, which are then used in oxidative phosphorylation to generate adenosine triphosphate (ATP). However, under anaerobic or high oxygen demand conditions (such as during exercise or inflammatory immune responses), there is a risk of hypoxia which disrupts mitochondrial function, potentially leading to cell death. To guard against this, metabolic adaptations shift from deriving ATP from oxidative phosphorylation to glycolysis alone, which increases lactate levels. As a major myokine and exerkine [32], lactate exerts diverse regulatory effects on redox biology, mitochondrial biogenesis and energy substrate utilization, lipolysis, and histone lactylation in healthy subjects.

**Fig. 1:**
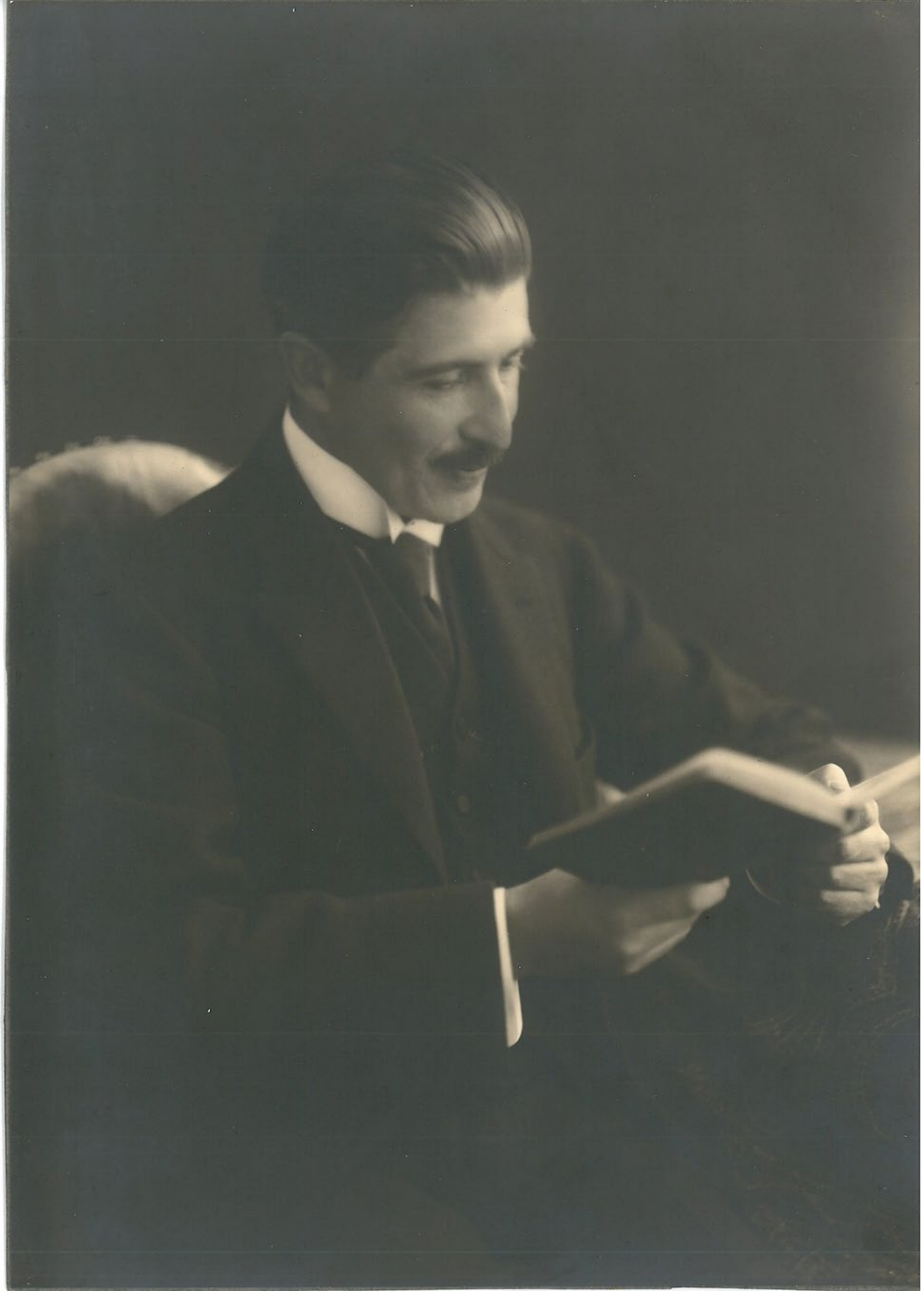
Prof. Edgard Zunz (1874–1939). Prof. Edgard Zunz was a prominent Belgian pharmacologist and the Director of the Laboratory of Pharmacodynamics and Therapeutics at the University of Brussels. Anaphylactic shock was one of Prof. Zunz’ research interests. In 1924, Prof. Zunz and his coworker Jean La Barre first reported lactate release during anaphylactic shock in their experiments with guinea pigs. In 1934, Prof. Zunz served as the President of the Belgian Royal Academy of Medicine. The portrait of Prof. Edgard Zunz was kindly provided by the Belgian Royal Academy of Medicine

Moreover, Zunz and La Barre (Fig. 1) showed that lactate (lactic acid) is centrally involved in anaphylactic shock in guinea pigs, thus marking 100 years of research in this area [33]. In the 1920s, Nobel Prize winners Otto Meyerhof [34] and Archibald Hill [35] demonstrated the role of lactate (lactic acid) in anaerobic conditions. During this period, Otto Warburg discovered that glycolytic activity, and subsequent lactate production, in cancer cells increases despite normoxic conditions, which is called the Warburg effect [36, 37]; it is still not fully understood a century later [38]. George Brooks [39] developed a lactate shuttle theory, demonstrating that lactate links glycolytic and oxidative metabolism [40]. Recognition of lactate shuttling came from physical exercise studies but now is being extended to other research areas. Over the last two decades, lactate has been increasingly recognized as a signalling molecule in various biological contexts [41], including chronic inflammation [42] and cancer [43].

Our interest stemmed from several mechanistic findings in lactate metabolism in sports immunology, intensive care science and immune reprogramming, pointing towards a role for lactate in different contexts, beyond cancer [42], especially in anaphylaxis. Since lactate is significantly associated with fatal anaphylaxis [31], it is crucial to determine whether its release fulfils the unmet need for more efficient biomarkers in the precision management of anaphylaxis [28] and other mast cell (MC)-dependent diseases [44].

Insights about lactate in anaphylaxis lie at the intersection of exercise immunology, critical care and allergy metabolomics. Lactate release is substantially, both locally and systemically, elevated during acute exercise and adaptations to chronic exercise, including increasingly popular endurance exercise. Lactate shuttles, described by George Brooks [39], highlighted lactate-mediated cell-to-cell crosstalk during exercise in healthy individuals. Since exercise is a known trigger or co-factor in anaphylaxis, this suggests a mechanistic relevance for lactate and associated metabolic changes during anaphylactic events, particularly in EIA.

In critical care settings, most evidence on lactate metabolism has come from studies in patients with various states of shock [45, 46], including a small number of studies on anaphylaxis [31, 47]. Higher lactate levels are associated with worse outcomes from anaphylaxis in a critical care setting [31]. Hence, lessons learned regarding lactate in critical care medicine may help predict and prevent fatal anaphylaxis. In allergy research, metabolic studies have focused mostly on lactate metabolism in asthma [48] and food allergy [49], including food allergic children with or without history of anaphylaxis [50]. In this regard, metabolic profiling of anaphylaxis is an area of considerable interest, though currently under-researched.

It is high time to shed light on these lactate research intersections between exercise biology, allergy metabolomics and shock pathophysiology and apply important lessons derived from them to anaphylaxis. Arguably, sufficient evidence has accumulated to evaluate the role of lactate in anaphylaxis. Therefore, in this review, we aimed to evaluate in vitro and in vivo evidence for lactate release from MCs, preclinical microdialysis studies, animal models and clinical data in anaphylaxis to glean novel insights, highlight clinical needs and inform the research agenda in this area.

## Role of Mast Cells in Anaphylaxis and Their Association with Lactate

MCs are key players in anaphylaxis owing to their capacity to release histamine and other vasoactive mediators following allergen provocation or via IgE-independent triggers such as anaphylatoxins [51] and Mas-related G-protein coupled receptor member X2 (MRGPRX2) [52, 53]. Detection of the MC-specific protease, tryptase, is an established indicator of systemic anaphylaxis [54], although its contribution in acute allergic reactions is poorly understood. Tryptase is often used as a surrogate for MC-specific activation where it is released in close correlation to histamine [54], elevations of which were also long known to be associated with EIA [55].

Interestingly, MCs also produce lactate in parallel to histamine release (HR) under normoxic conditions [56–72] (Supplementary Table S1). However, this has only been shown in vitro in rodent mast cells and we simply do not know the proportions of lactate from MC and other sources contributing to its systemic release in humans. MCs depend on glycolytic and oxidative pathways to mitigate their energetic expenditure during degranulation [73, 74]. The signalling pathways underlying lactate release during MC degranulation involve hypoxia-inducible factor-1α (HIF-1α) stabilization, which crucially upregulates glycolytic enzyme expressions (e.g. LDH-A), and suppresses the tricarboxylic acid (Krebs) cycle. HIF-1α expression was shown to increase following FcεRI-mediated stimulation of human MCs as well as basophils, where it plays a crucial role in enabling these cells to generate inflammatory and immunomodulatory cytokines within hours following degranulation [75]. HIF-1α stabilization is a critical step in enhancing glycolytic pathways, which prevent potential adenosine trisphosphate (ATP) depletion, in stimulating angiogenesis, including the production of vascular endothelial growth factor (VEGF), and in the subsequent formation of lactate. HIF-1α accumulation was further shown to be dependent on the mammalian target of rapamycin (mTOR) kinase [76], a central regulator of myeloid cell growth and metabolism, which was recently shown to play a vital role in the ability of MCs to re-granulate following activation [77].

The immunometabolism of allergic effector cells, including mast cells and basophils, relies on glycolysis upon acute activation (the Warburg effect) to address a high energy demand for their activation [75, 78, 79]. Their rapid release of preformed mediators, such as histamine, as well as de novo*-*generated lipid and cytokine mediators, increased demands in ATP generation (used for the phosphorylation of kinases and other signalling proteins). These cells shift towards producing lactate from pyruvate, which is mediated via HIF-α-dependent upregulation of different pyruvate dehydrogenase kinases. Although aerobic glycolysis is less energetically efficient than oxidative phosphorylation, the former allows a faster energy generation under the circumstances of rapidly increased energy demands for immune cell activation. Indeed, IgE-mediated activation of basophils was shown in vitro to dose-dependently decrease ATP levels while increasing HIF-1α accumulation [75, 79]. The resulting induction of glycolysis and glucose transporters subsequently protects the cells against ATP depletion and allows them to fulfil their effector functions.

The energetics of MC degranulation are incompletely understood [74]. Early studies suggested a link between ATP levels and HR, where inhibition of glycolysis blocked HR but did not affect ATP production [66, 80]. Being an energy source [39], lactate may be required for MC energetics since lactate production increases when ATP and oxygen demands exceed supply [81]. Whether lactate contributes to energy metabolism during MC degranulation can be studied by isotope-tracer studies in animal models of anaphylaxis. Furthermore, genetically encoded fluorescent biosensors for extracellular [82] and intracellular lactate [83] and single-cell metabolomics [84] may address these questions in MC lines in future studies.

Net lactate production increases under various conditions, including exercise or inflammation, and elevated lactate concentrations may affect immune cell functions, including MCs [85]. Extracellular lactate is transported across the plasma membrane by four l-lactate-transporting monocarboxylate transporters (MCTs) [86]. In MCs, extracellular lactate may enter MCs by the ubiquitously expressed MCT1 (also known as SLC16a1 [86]), which has a high affinity for lactate (*K*_m_ = 3.5–10 mM) [42]. CD147, a transmembrane glycoprotein which co-localizes with lactate transporters MCT1, MCT3 and MCT4 [86], was reported in skin MCs [87], although lactate transporters and CD147 expressions have not been evaluated in anaphylaxis.

**Fig. 2:**
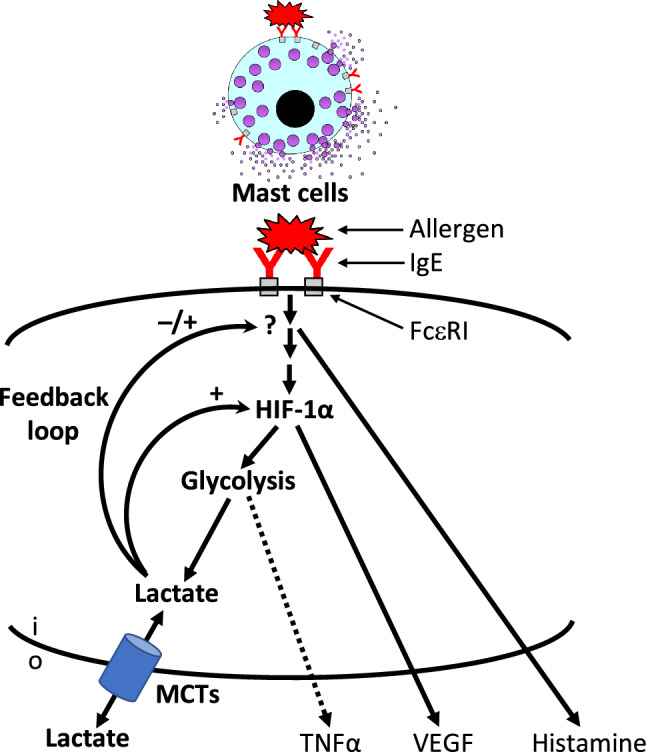
Overview of the HIF-1α-mediated production of lactate in mast cells. Activation of MCs and related allergic effector cells (e.g. basophils) leads to HIF-1α stabilization which regulates the production of VEGF and induces glycolysis. Glycolytic pathways generate lactate as well as preventing ATP depletion in activated MCs, thereby facilitating de novo-generated cytokine production. Lactate is subsequently released from mast cells and may affect MC responses, suggesting a potential negative feedback loop which remains to be elucidated in anaphylaxis

Functional studies primarily focused on lactic acid (2-hydroxypropionic acid, C_3_H_6_O_3_) rather than lactate (2-hydroxypropanoate, C_3_H_5_O_3_^−^), where inhibitory effects on MCs have been demonstrated in mechanistic studies [68–72]. Syed and coworkers [72] reported that lactic acid suppressed MRGPRX2-mediated MC activation in LAD2 MCs, human skin MCs and mouse peritoneal MCs. Caslin and coauthors [70] demonstrated that lactic acid inhibits LPS-induced MC function by limiting glycolysis and ATP availability. Lactic acid suppressed IgE-mediated mast cell responses in vitro and in vivo, suggesting a role for lactate as a negative feedback loop which limits MC activation (Fig. 2) [69]. However, only moderate inhibitory effects were observed with lactate compared with lactic acid [69, 70, 72], suggesting an essential role of pH; it remains uncertain whether these effects can be induced by lactate in vivo [88]. Despite this, Abebayehu et al. [69] showed that the inhibitory actions of lactic acid on MC function were dependent on MCT1, underlining a role for a lactate/lactic acid-specific effect rather than H^+^ generated from other sources.

The potential role of lactate as a signalling molecule in MCs clearly requires further study. One could speculate that lactate signalling in MCs may be abnormal in MC-dependent conditions, including chronic spontaneous urticaria, chronic inducible urticarias (CINDU) or EIA, especially in terms of potential inhibitory feedback loops as implied by previous in vitro studies [69, 70, 72]. However, the notion that lactate has such inhibitory effects on MCs is largely based on observations using lactic acid rather than lactate per se. Whether lactate accumulation in reported patients [31] is due to excessive production or impaired oxidative metabolism also remains to be elucidated.

MC activation is central to anaphylaxis [51]. However, other effector cells (neutrophils, platelets, basophils, macrophages and monocytes) also contribute to its pathophysiology [89]. Importantly, MC activation may indirectly increase lactate levels owing to the actions of histamine on the endothelium. Ziogas et al. recently demonstrated that histamine stimulates hyperpermeability in human microvascular endothelial cells (H1-receptor mediated) by triggering phospholipase C and glycolysis, subsequently significantly enhancing lactate levels [90]. Furthermore, these histamine-induced effects were abrogated by the glycolytic inhibitor 3-(3-pyridinyl)-1-(4-pyridinyl)-2-propen-1-one (3PO), which in vivo crucially protected mice from developing passive cutaneous as well as active systemic anaphylaxis.

The above clearly provides a plausible explanation regarding the association between increased lactate levels in MC-related diseases, due to their histamine-releasing capacity. The release of this biogenic amine from basophils in the circulation is also implicated. Moreover, increased glycolysis associated with ongoing inflammation or exercise may reduce the threshold and subsequently potentiate the severity of histamine-induced vascular permeability. If this is the case, then histamine releasability and/or the polymorphisms and expression levels of H1 receptors on endothelial cells of, for example, patients with anaphylaxis, may be important. Finally, tentative evidence suggests that MC-derived histamine plays a physiological role in optimizing muscle perfusion via H1 and H2 receptors [91, 92]. While MC activation is clearly associated with acute aerobic exercise (as determined by elevated tryptase levels) [91], an important known unknown is how MCs are activated in exercise per se.

**Fig. 3:**
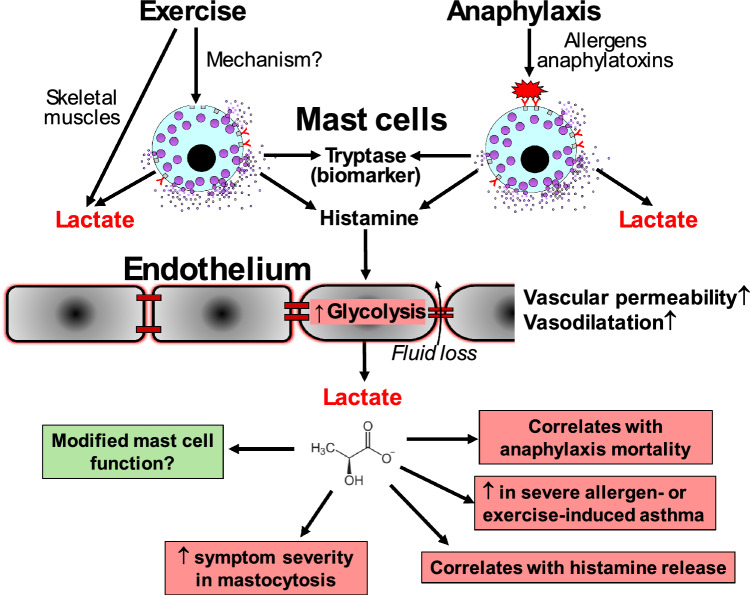
Overview of the differential sources and role of lactate in exercise and anaphylaxis. In addition to its release from skeletal muscles during exercise, lactate is also released from mast cells potentially both during exercise (through yet unknown mechanisms) and in anaphylaxis (following activation by allergens or anaphylatoxins). Histamine is also released from mast cells and stimulates endothelial cells, which results in further lactate release due to the activation of glycolytic pathways. The resulting increased vasodilatation, vascular permeability and subsequent fluid caused by the effects of histamine on the endothelium substantially contributes to the signs and symptoms of anaphylactic shock. The association with lactate release in limited clinical data and activated mast cells in in vitro data suggests that lactate may be a potential indicator or biomarker of mast cell activation. However, lactate may also have regulatory effects on mast cells in some settings, which needs to be further investigated

There is, therefore, a commonality of MC activation in both exercise and anaphylaxis, which, though orchestrated by different stimuli, results in increased MC mediators and lactate. This notion is further supported by the findings of Parsons et al. [93], showing that post‑exercise hypotension and exercise-associated collapse is also dependent on MC degranulation in marathon runners. The severity of anaphylaxis and other reactions related to exercise hypersensitivity is thus likely to involve thresholds of sensitivity to histamine, and other MC-derived mediators, which are released both physiologically and due to allergic mechanisms, both of which are associated with elevated lactate (summarized in Fig. 3). It remains to be established whether endurance exercise, which is a known hyperglycolytic state, induces glycolytic processes in MCs, vascular endothelium and intestinal epithelial cells, thus affecting the end-organs of immune mediators in anaphylaxis. These aspects, together with other diverse signalling contributions of lactate in health and disease [42, 43], highlight the need to understand this important metabolic messenger, a ‘lactormone’ [39, 94], in the context of anaphylaxis in greater detail.

## Preclinical Studies in Humans and Animal Models

### Preclinical Studies in Animal Models

Since the seminal work by Paul Portier (1866–1962) and Charles Richet (1850–1935) first on the effects of *Physalia physalis* (Portuguese man-of-war) toxins on various small animals during a cruise on the yacht Princesse Alice II of Prince Albert of Monaco in the summer of 1901, and then their definitive experiments with dogs sensitized with *Actinia* extracts [95], there have been numerous animal models of anaphylaxis [96]. Lactate release was reported within 30–60 min in anaphylaxis in dogs [97], rabbits [98] and mice [99, 100]. Despite limitations related to species-specific differences and animal genetic backgrounds [96, 101], these animal models may provide further insights into lactate metabolism in anaphylaxis.

Many currently unresolved questions about lactate in anaphylaxis, such as the cellular sources, the kinetics and the catabolism in anaphylaxis, may be, at least partly, answered in isotope studies, with or without exercise or allergen challenges, in mouse or rodent models of food-induced anaphylaxis, including a humanized mouse model of peanut anaphylaxis [102–104].

### Preclinical Studies in Humans

Microdialysis [105, 106] has been used in metabolic studies [107] in the skin and skeletal muscles in humans to study lactate metabolism at the tissue level. Except the study by Rosdahl et al. [108], baseline lactate concentrations in healthy subjects were two- to threefold higher in the skin and skeletal muscles than those in venous blood [107, 109, 110], underlining that local lactate release can be a promising readout for microneedles and wearables. Importantly, lactate–pyruvate ratios in dermal dialysates indicated normoxic conditions [109, 110], which is in keeping with a contemporary view of lactate production under fully aerobic conditions [39]. Although the skin, particularly the epidermis [111], produces large amounts of lactate on a par with resting skeletal muscles in humans [46, 112], cellular sources of dermal lactate release are unknown [107]. Recent metabolomic study of skin dialysates in healthy subjects demonstrated that lactate levels were fourfold higher in skin dialysates compared with peripheral blood [113]. Further metabolomic cutaneous microdialysis studies in exercise are underway in Japan.

Interstitial lactate levels in skeletal muscles were shown to rise during intermittent static [108] or dynamic exercise [114], consistent with the lactate shuttle hypothesis [39]. Kinetic studies in healthy subjects demonstrated that tissue lactate concentrations remain elevated for an hour (skeletal muscles) following exercise [108] and for 90 min in the skin following oral glucose tolerance tests [107]. Such mechanistic studies now need to be applied to patients with anaphylaxis, using microneedles and wearable biosensors [115] to monitor cutaneous lactate levels in the context of controlled challenges (exercise, allergen or both).

## Clinical Evidence for a Role of Lactate in Anaphylaxis

### Adaptive Metabolic Response to Exercise

The time course of exercise-induced molecular events is of particular interest given that most EIA cases occur within 1 h (mostly 10–30 min) after exercise [116, 117]. Lactate is known to mediate acute response and long-term adaptations to exercise [118]. Lactate production occurs in fast-twitch white skeletal fibres and in populations of red fibres at the start of exercise [119]. In 30-min endurance tests, there was a significant increase in blood lactate concentration for both trained and untrained individuals [120]. A recent systematic review highlighted increased MCT1 and, to a lesser extent, MCT4 expressions in skeletal muscles following a training program [121].

In skeletal muscles, the peroxisome proliferator-activated receptor-γ coactivator 1α (PGC-1α) alters the LDH complex, thereby controlling lactate homeostasis and skeletal muscle metabolic adaptations in response to exercise. In a meta-analysis of human transcriptomic studies, *PPARGC1A* expression, a central transcriptional regulator of skeletal muscle adaptation to exercise, was upregulated in skeletal muscle biopsies after acute aerobic and resistance exercise [122]. In a human exercise transcriptome study [123], *NR4A1* mRNA was increased after aerobic exercise (1 h), whereas mRNA for transcription factors *HIF1A* and *PRARGC1A* was increased at 3 h post exercise. Therefore, in healthy individuals, adaptive responses of lactate metabolism to exercise comprise increased lactate in both blood and skeletal muscles, followed by upregulated signalling pathways (NR4A1, HIFα, and PGC-1α) and altered LDH complex and lactate transporter expressions.

Adaptive metabolic responses to acute exercise in humans also include HR [124] as was first reported in healthy subjects following physical exercise on a bicycle ergometer in the 1950s [125]. HR is detected at 30–60 min during exercise. Although histamine is usually associated with allergic and anaphylactic responses, this mediator is also released in the context of exercise adaptations. In healthy subjects, acute aerobic exercise for 1 h induces a twofold upregulation of histidine decarboxylase (HDC) mRNA 3 h following exercise [123]. Aerobic exercise also induces a post-exercise activation of histamine H1 and H2 receptors [123], and these receptors were shown to mediate vasodilatation associated with post-exercise hypotension [126].

The involvement of MCs is further supported by the findings of Parsons et al. [93], showing that post‑exercise hypotension and exercise-associated collapse is also dependent on MC degranulation in marathon runners. In the study by Parsons et al. [93], marathon runners with or without exercise-induced hypotension or exercise-associated collapse demonstrated tryptase release following a marathon race, clearly indicating MC involvement in exercise-induced hypotension per se. Runners with a history of exercise-induced hypersensitivity syndromes were excluded, and a personal or family history of atopy was not evaluated in this study. Therefore, exercise adaptations of histamine metabolism include HR in skeletal muscles and, variably, in the circulation, as well as upregulation of HDC and activation of histamine receptors, all of which are important in anaphylaxis.

### Relation to Exercise as a Trigger in Anaphylaxis

In a clinical context, multiple lines of evidence suggest that exercise is a common trigger in anaphylaxis, including food-dependent exercise-induced anaphylaxis (FDEIA) [22]. On the basis of the data of 7316 patients from the Anaphylaxis Registry, patients who reported vigorous exercise during (or prior to) a given reaction had a higher risk [odds ratio (OR) 1.5, 95% confidence interval (CI) 1.3–1.7] of severe anaphylaxis compared with those not physically active [127]. Exercise may also trigger certain chronic inducible urticarias (CINDUs), such as cholinergic urticaria [128], which can be associated with anaphylaxis. Moreover, in adults allergic to peanuts, exercise has been shown to reduce threshold reactivities to oral challenge [21], suggesting that exercise may play a role in conventional anaphylaxis too, at least in certain individuals. Exercise is known to increase allergen absorption in the gut [129] which, at least in part, offers a credible explanation for reduced reactivity thresholds to allergens exposed via the gastrointestinal tract as is the case with FDEIA and other severe food allergies. However, the physiological release of histamine in exercise may serve as an additional parameter for reduced symptom severity thresholds by acting in concert with allergen-induced HR and its downstream actions. This, for example, could further enhance endothelial permeability and therefore exacerbate crucial mechanisms which govern anaphylaxis severity.

EIA is a rare and potentially fatal condition in which anaphylaxis occurs during or after exercise, without exogenous allergen stimulation [130]. Importantly, various CINDUs were reported in athletes, mostly in those practicing field and track sports [131]. Potential mechanisms underlying EIA include altered gastrointestinal permeability, increased transglutaminase activity in the gut mucosa, blood flow redistribution, changes in plasma osmolality and subsequent basophil HR, as well as cellular pH changes leading to MC degranulation [130].

### Exercise-Induced Anaphylaxis: Lactate and MC Degranulation

Since its description in an endurance runner in 1979 [116], there have been up to 800 cases reported in literature [127], with many patients being established athletes [132]. Exercise-induced cutaneous MC degranulation was reported in patients with EIA [133]. Laboratory treadmill challenges can provoke the symptoms in patients with EIA, although negative challenges were also reported [134]. Of interest, responders to exercise challenge had a statistically high mean increase from baseline levels of histamine (tenfold) compared with non-responders [134]. In EIA responders undergoing exercise challenges, HR was noted to increase within 10 min [135] and to subside within 20–60 min after cessation of exercise [136, 137]. Exercise-induced tryptase release was demonstrated in an 18-year-old football player with EIA [137]. The mechanisms of MC activation in EIA are unknown. The data on systemic HR in exercise challenges were rather inconsistent in patients with EIA [138], possibly due to its short half-life and technical issues with detection techniques [139]. The latter are likely to be resolved with the advent of metabolomics and biosensing. Anaphylactic responses to acute exercise in patients with EIA during a controlled challenge are known to be linked to MC degranulation and systemic histamine/tryptase release [133, 134, 137]. EIA pathophysiology is likely to result from interplay between the type and intensity of exercise, the athletic history of the patients, adaptations of lactate and histamine metabolisms, and activation thresholds for basophils and MCs, which are the main sources of histamine.

As an important exerkine [32, 140], lactate may play a role in exercise-related, MC-dependent conditions, such as in EIA, which commonly occurs in atopic patients [134] and/or athletes, including endurance athletes [141, 142]. Clinical evidence of exercise-induced lactate release in MC-dependent diseases is limited to a case report with exercise-induced urticaria/angioedema [136]. In a 21-year-old patient with exercise-induced urticaria and angioedema, lactate concentrations rose to high levels (13 mmol/L), accompanied by HR, following treadmill challenge [136]. Further studies are needed to elucidate whether metabolic exercise adaptations, including lactate and HR, augment the pathophysiological mechanisms of EIA.

The tissue crosstalk between the skin and exercising muscles mediated by lactate, as proposed by Brooks [143], indicates that lactate may mediate tissue-to-tissue communication during exercise in health and in MC-dependent conditions. However, the contribution of lactate, released during physical exercise, to the pathophysiology of EIA has been given insufficient attention. There is a need for comparative metabolomic studies in healthy subjects and patients with EIA since it is unclear whether hyperlactatemia enhances HR from MCs in exercise-induced hypersensitivity syndromes as suggested by Tse and co-workers [136]. Whether adaptive and anaphylactic responses to exercise overlap in EIA or whether exercise adaptations create permissive conditions for EIA is also unclear.

### Exercise-Induced Urticaria: Lactic Acid and Skin Testing

Interestingly, in 1949 Herlitz [144] reported that lactic acid produced a wheal-and-flare response in two patients with exercise-induced urticaria. These reactions were transferable by the Prausnitz–Küstner test, suggesting that, in rare cases, the lactate molecule itself may either activate MCs or affect MC activation thresholds in urticaria. However, this observation of potential stimulatory effects of lactic acid contrasts with more recent findings regarding an inhibitory action of lactic acid on MCs [69, 70, 72], underlining an urgent need for further studies.

### Anaphylaxis in Mastocytosis

Patients with mastocytosis are at high risk of severe anaphylaxis [145]. Anaphylaxis can occur in 20–56% of adult patients with mastocytosis owing to excessive MC mediator release [146]. Preventive management of anaphylaxis in mastocytosis includes antihistamines, MC stabilizers, life-long immunotherapy in patients allergic to Hymenoptera venom, cytoreductive therapies for patients with an excessive MC burden [145] and targeted therapies for patients with frequent recurrent anaphylaxis [146].

In mastocytosis, maximal clinical symptoms during a severe MC activation event were strongly associated with both lactate and histamine levels (Supplementary Table S2) [147], supporting previous observations of MC lactate release (Supplementary Table S1). Here, lactate rose to 7 mM at the peak of the attack, with a rapid decline at 1.1 mM/h with a half-life of 153 min [147], exceeding the usual half-life of lactate post-exercise. Future studies need to establish whether metabolomic measurements, including lactate, and using wearable biosensors (Graphical Abstract) can help diagnose and manage such recurrent anaphylactic events.

### Relation to Anaphylaxis Severity

In a multi-centre French study of 339 patients with anaphylaxis in an intensive care setting, lactate release was shown to be associated with fatal outcome by multivariate analysis [31]. Although not specific to anaphylaxis, the levels of lactate in non-survivors of anaphylaxis were higher than those observed in septic or cardiogenic shocks [148, 149]. Importantly, future studies need to evaluate changes in lactate concentrations from the onset of symptoms rather than from the time of admission to a critical care unit to truly assess how lactate levels evolve in anaphylaxis. Although this may be challenging for food-induced anaphylaxis in an out-of-hospital setting, this should be imminently possible for perioperative anaphylaxis.

In the study by Crestani et al. [50], non-targeted global metabolomics revealed significantly different lactate levels in food-allergic children with (*n* = 19) or without (*n* = 51) a history of anaphylaxis, requiring intramuscular epinephrine injection. Using plasma metabolomics, altered carbohydrate metabolism, including decreased levels of pyruvate together with increased levels of lactate, was observed in patients with severe food allergy to profilins, including three patients who required administration of epinephrine [150]. In addition, on the basis of gene set enrichment analysis (GSEA), oxidative phosphorylation was reduced in profilin-allergic patients with severe manifestations on oral challenge, thus suggesting a switch to Warburg metabolism [150].

Metabolomic profiling (Supplementary Table S2) demonstrated metabolic differences during the acute reaction (< 2 h) and following clinical recovery (2–4 h later) in anaphylaxis [47]. In the acute phase, 32.62% patients with moderate anaphylaxis had increased lactate levels, compared with a recovery phase, demonstrating a decline in lactate concentrations in these patients following a recovery. The present evidence supports a role for elevated lactate levels in predicting anaphylaxis severity. Interestingly, patients with severe anaphylaxis had higher lactate levels 2–3 months later compared with those with moderate anaphylaxis [47]. Whether this reflects hyperglycolytic changes and/or MC hyperreleasability associated with severe anaphylaxis is currently unknown.

### Relation to Epinephrine Treatment

Under physiological conditions, elevations in lactate production are a known metabolic effect of epinephrine in healthy individuals during exercise [151]. Previously, metabolic effects on lactate levels associated with epinephrine (epinephrine-induced hyperlactatemia) were shown to be mediated via β_2_ adrenergic stimulation of muscle tissue Na^+^/K^+^-ATPase-activity [152].

Raised lactic acid to high lactate-to-pyruvate ratios in arterial blood and elevated serum lactate dehydrogenase (LDH) levels were described in two patients with anaphylaxis, both treated with epinephrine [153]. Lactic acidosis was also reported following epinephrine injections in a patient with anaphylaxis [154]. Given that around one in ten anaphylactic episodes are treated with multiple epinephrine doses [155], epinephrine-induced lactic acidosis may present a clinical challenge, and an unmet need for therapeutic strategies, requiring serum lactate monitoring during critical care. If epinephrine-induced lactic acidosis is suspected, alternative vasopressors should be considered.

## Lactate as a Potential Biomarker of Anaphylaxis: A Critical Appraisal

Existing biomarkers, e.g. serum tryptase, histamine, and prostaglandins in urine or skin testing, may be used in clinical practice but not as bedside biomarkers [30]. Since anaphylaxis severity is impossible to predict [156], lactate and associated metabolic changes may unlock this present impasse, where lactate measurements, using disposable biosensors and wearables, offer potential point-of-care testing (Box 2) [157–161]. Understanding the advantages and pitfalls of lactate as a putative anaphylaxis biomarker holds a promise for personalized anaphylaxis management (Supplementary Table S2).

**Box 2:** Clinical lactate measurements

**Healthy subjects at rest**
Lactate concentrations in peripheral blood and dermal interstitial fluid (ISF) in healthy individuals are within the range of 1–2 mM Hyperlactatemia is defined as serum lactate level of 2 mM or greater [140] **Healthy subjects during exercise** At sea level, lactate levels > 4 mM are frequently detected in training [137] and can increase up to ≈ 15–25 mM in intense exercise [138] **Critically ill patients** Lactic acidosis is characterized by elevated lactate levels (> 5 mM) and pH < 7.35 [132–140, 142] In patients with severe sepsis and septic shock, hyperlactataemia can raise up to 15 mM **Patients with anaphylaxis** Epinephrine-induced lactic acidosis may occur [134] **Lactate biosensing** Multiple **biosensors** have been developed for lactate measurements in peripheral blood and dermal interstitial fluid, with a low limit of detection (< 0.1 mM) and a wide linear range (0.1–100 mM). A clinical trial of a lactate biosensor is ongoing (ClinicalTrials.gov no. NCT04238611)

Lactate is an easy-to-measure parameter, thus fulfilling a requirement that biomarkers must be measurable [162, 163]. However, several limitations may hinder its development as a biomarker. The main challenge is the lack of an interpretation framework of lactate concentrations in anaphylaxis. Lactate is reflective of complex metabolic changes and can be elevated in the context of any critical illness but not specifically in anaphylaxis.

Lactate metabolism not only plays a role in anaphylaxis but also in atopic diseases such as atopic dermatitis (AD) and asthma. LDH activity (Supplementary Table S3) is substantially upregulated in in AD [164, 165]. Likewise, elevated lactate was reported in patients with asthma, including those with status asthmaticus [166]. A more recent study underlined that increased lactate is associated with asthma pathogenesis and is crucially associated with increased glycolysis [48], suggesting that it could serve as a biomarker for asthma severity. In the study by Ruman-Colombier and co-workers [167], 87% of patients hospitalized with moderate and severe asthma had hyperlactatemia, with lactate concentrations exceeding 5 mmol/L in 26% of patients. This indicates that lactate is already involved in these atopic diseases; hence, the diagnostic value of lactate measurements in patients with anaphylaxis with comorbid atopic diseases needs further research.

On balance, lactate may be an attractive candidate for biomarker development in anaphylaxis. Although histamine and lactate are both released from MCs, histamine is known to induce a hyperglycolytic state in vascular endothelium which, in turn, leads to further lactate release [88]. Thus, its significance as biomarker, especially as an indicator of vascular involvement in EIA remains to be proven. Whether lactate measurements may offer advantages over the established biomarker tryptase are yet to be demonstrated. In the prospect, this may lead to a development of an integral biomarker signature in anaphylaxis, including lactate, reflecting different pathways and pathogenic steps in anaphylaxis.

## Summary and Perspectives

While not causative or specific to anaphylaxis per se, lactate is involved in all steps of anaphylaxis, including MC degranulation and vasodilatation. However, lactate may be considered a potential candidate for biomarker development in anaphylaxis, in combination with other known biomarkers. The overarching notion that lactate is involved in anaphylaxis, and especially its severity, is supported by decades of evidence indicating its mechanistic and clinical relevance, albeit one that has not been systematically addressed or fully understood. Lactate may emerge as a potential missing link between anaphylaxis, MC function, and exercise, underlining an intriguing commonality of mechanisms which have so far been largely overlooked.

This perspective raises the question whether the release of lactate and histamine in anaphylaxis is entirely independent or a one-directional event, reflective of hyperglycolytic states and MC degranulation and whether there are amplifying or inhibitory relationships between these mediators. In our opinion, the most relevant in vitro finding is that histamine induces lactate release from endothelial cells, suggesting that lactate release in anaphylaxis can be induced, at least in part, by histamine. Another important question is whether hyperglycolytic states (Supplementary Box S1) in anaphylaxis and other allergic or urticarial diseases may open new therapeutic possibilities. However, many crucial questions regarding the role of lactate in anaphylaxis remain to be addressed (summarised in Table 1).

**Table 1:** Unanswered questions in lactate research in anaphylaxis

	Category	Unanswered questions
1	Aetiology	Do lactate levels differ depending on the aetiological factor of anaphylaxis?
		What are the lactate levels in (food-dependent) exercise-induced anaphylaxis or anaphylaxis associated with CINDUs?
		What are the lactate levels in anaphylaxis with exercise as a cofactor?
2	Epidemiology	Can lactate measurements at rest or during exercise detect individuals who are at risk of anaphylaxis?
		Are endurance athletes more likely to develop urticaria/anaphylaxis?
3	Pathophysiology	What is the role and relevance of lactate in the pathogenesis of anaphylaxis?
		Do lactate levels and release kinetics differ depending on the anaphylaxis endotypes?
		Does lactate release differ in IgE-dependent and non-IgE-dependent anaphylaxis?
		Does lactate contribute to MC regulation in anaphylaxis? If so, how?
		Is the MC lactate signalling dysregulated in anaphylaxis?
		What is the expression level of lactate receptors on MCs in patients with anaphylaxis?
		Are MCs, endothelial cells and gastrointestinal epithelium in a hyperglycolytic state in endurance athletes?
		What are the overlap mechanisms between exercise adaptations and priming in atopic diseases?
		What are the local events in the skin underlying lactate release in healthy subjects and in patients with anaphylaxis?
4	Clinical heterogeneity	Do metabolic signatures differ between anaphylaxis phenotypes?
		What are the lactate levels in different anaphylaxis phenotypes?
		Is there a difference in lactate release between exercise-induced anaphylaxis and other exercise-induced urticaria/angioedema?
		At what lactate levels does fatal or near-fatal anaphylaxis occur?
		What is the lactate release pattern in anaphylaxis associated with CINDUs?
5	Comorbidities	What is the shared lactate pathophysiology of anaphylaxis with comorbid atopic diseases?
		What are the characteristics of lactate metabolism in patients with anaphylaxis with comorbid skin diseases?
		What are the characteristics of lactate release in patients with anaphylaxis with or without comorbid atopic diseases?
		What are the mechanisms underlying lactate release in atopic and non-atopic patients with anaphylaxis?
6	Clinical course	What are the lactate levels in patients with severe anaphylaxis?
		What are the lactate levels and kinetics in patients with exercise-induced anaphylaxis?
		What is the metabolomic signature of life-threatening or fatal anaphylaxis?
		What are the predictive models for the risk of anaphylaxis and for the disease severity in anaphylaxis associated with CINDUs?
		What is the accuracy of lactate as the prognostic biomarker in exercise-induced anaphylaxis?
7	Diagnosis	Can lactate release pattern be useful for severity grading in anaphylaxis?
		What are the local events in the skin underlying lactate release in atopic and non-atopic endurance athletes?
		Can the local lactate release in the skin be measured in individuals with a history of anaphylaxis using wearables?
		Can the measurements of skin lactate release, using wearables, detect early signs of anaphylaxis?
8	Laboratory testing	What is the clinical relevance of lactate measurements in patients with anaphylaxis?
9	Differential diagnosis	What is the impact of metabolomics on the differential diagnosis of anaphylaxis?
		Can we differentiate anaphylaxis and exercise-induced hypotension using lactate and metabolomic signature?
10	Treatment	Can lactate levels guide the treatment of anaphylaxis?
		Can lactate levels inform clinicians about the efficacy of epinephrine?
		What is the lactate release pattern following epinephrine injection in patients with anaphylaxis with or without lactic acidosis?
		Can lactate be a predictive biomarker for treatment efficacy in anaphylaxis?
		Can lactate be a novel therapeutic target in anaphylaxis?
		What are the risk factors for epinephrine-induced lactic acidosis in patients with anaphylaxis? Are patients with multiple epinephrine injections at risk of lactic acidosis?
		What is the optimal management of patients with anaphylaxis with epinephrine-induced lactic acidosis?

CINDU, chronic inducible urticarias

Finally, exercise is associated with both lactate and HR, posing novel questions as to how physiological and anaphylactic responses to exercise can be bridged in EIA. These questions have been in the field for a hundred years and have not been resolved. Yet, they must be resolved given the mechanistic plausibility, expanded knowledge of lactate metabolism, advent of lactate biosensors and accumulation of clinical evidence regarding the life-threatening condition of anaphylaxis.

## Glossary

Aerobic glycolysis: under aerobic conditions, 1 mole of glucose is metabolized to pyruvate, which, in turn, is converted to acetyl-coenzyme A, which, via tricarboxylic acid (Krebs) cycle, is oxidized to carbon dioxide, with a production of 38 moles of ATP [159].
Anaerobic glycolysis (Embden–Meyerhof pathway): under anaerobic conditions, pyruvate is converted to lactate via the Embden–Meyerhof (glycolytic) pathway with a production of only 2 moles of ATP per mole of glucose [159].
Anaphylaxis: a serious allergic reaction that is rapid in onset and potentially life-threatening. Anaphylaxis is highly likely when any of the following three criteria are met:
  1. Acute onset of illness within minutes to hours, with involvement of the skin or mucosal tissue and at least one of the following: respiratory compromise, reduced blood pressure, or symptoms of end-organ dysfunction; or
  2. Two or more of the following that occur rapidly (minutes to hours) after exposure to a likely allergen or other trigger for that patient: skin or mucosal tissue involvement, respiratory compromise, reduced blood pressure or associated symptoms, or gastrointestinal symptoms; or
  3. Reduced blood pressure^*^ that occurs within minutes to hours after exposure to a known allergen for that patient [3, 7].
  ^*^Low systolic blood pressure (age-specific) or greater than 30% decrease in systolic blood pressure in infants and children, whereas the systolic blood pressure is less than 90 mm Hg or there is a greater than 30% decrease from the patient’s baseline.
Biomarker: a characteristic that is objectively measured and evaluated as an indicator of normal biological processes, or pharmacologic response to a therapeutic intervention (National Institutes of Health Biomarkers Definitions Working Group [162]). For a biomarker, the relation to clinical indices or outcomes is important.
Biosensor: an analytical device that uses a biological sensing element and a physical transducer to produce an electrochemical, optical, mass or other signals, which together relates the concentration of an analyte to a measurable electrical signal.
CD147 (also known as Basigin, neurothelin or EMMPRIN (extracellular matrix metalloproteinase-inducer)): a transmembrane glycoprotein which co-localizes with lactate transporters MCT1, MCT3 and MCT4 and enhances their activities.
Cori cycle: a metabolic pathway, named after Carl Ferdinand Cori and Gerty Cori, that generates glucose (gluconeogenesis) from lactate in liver and kidney.
Exerkines: a broad variety of signalling moieties that are released in response to acute and/or chronic exercise that exert their effects through endocrine, paracrine and/or autocrine pathways [140].
HIF-1α: the inducible subunit of hypoxia-inducible factor 1 (HIF-1α) transcription factor, which crucially stimulates glycolysis, angiogenesis and cell adhesion.
Histamine: inflammatory mediator which plays a crucial role in eliciting the major signs and symptoms of acute allergic reactions, including anaphylaxis. Mast cells and basophils (which share many functional properties with mast cells but are fewer in number) are the principal sources of this biogenic amine. Its contribution to anaphylaxis includes stimulation of H1 receptors, which are involved in bronchoconstriction, vasodilatation, increasing vascular permeability and itch. H1 and H2 receptors are also expressed in the heart and play a major role in cardiac arrhythmias associated with anaphylactic shock. H4 receptors are also involved in pruritus (together with H1).
Histidine decarboxylase: an enzyme primarily expressed in mast cells and basophils involved in the generation of histamine by catalysing the decarboxylation of histidine.
Hyperlactatemia: elevated blood lactate concentration (> 2 mmol/L).
Krebs cycle (the tricarboxylic acid cycle): an aerobic metabolic pathway that produces 36 molecules of ATP for 1 molecule of pyruvate.
Lactate: a hydroxy monocarboxylic acid anion (the conjugate base of lactic acid), with two stereoisomers, produced during glycolysis
Lactate clearance: a function of production and uptake of lactate, mostly by liver and kidney, followed by metabolism that leads to a given serum lactate concentration. In clinical practice, lactate clearance refers to the changes in lactate concentrations over time.
Lactate dehydrogenase (EC 1.1.1.27): a tetrameric enzyme, with five isoforms, that acts as a catalyst in the reaction: pyruvate + NADH + H^+^ ↔ lactate + NAD^+^. LDH is composed of two subunits LDH-A and LDH-B, which belongs to the 2-hydroxyacid oxidoreductase family. LDH-A, characterized by a higher affinity for pyruvate, preferentially converts pyruvate to l-lactate, whereas LDH-B has higher affinity for lactate and catalyzes l-lactate to pyruvate [40]. LDH also regenerates NAD^+^ from its reduced form (NADH).
Lactate shuttle: a concept, developed by George Brooks [40], that describes the role of lactate in delivery of oxidative and gluconeogenic substrates, as well as in cell signalling.
Lactate-to-pyruvate ratio: a molar ratio that reflects the equilibrium between product and substrate of the reaction catalysed by lactate dehydrogenase.
Lactic acid (2-hydroxypropanoic acid): an organic acid belonging to the family of carboxylic acids (lactate is the conjugate base of lactic acid).
Lactic acidosis: a cellular metabolic process characterized by rises in blood lactate (> 5 mmol/L) and decreases in blood pH (< 7.25).
Lactylation: a newly discovered lactate-dependent metabolic reprogramming that involves a post-translational modification of histone lysine residues and impacts cell metabolism and function [41].
Mast cell degranulation: process involving the fusion of MC granules and rapid release, withing minutes, of preformed inflammatory meditators (e.g. histamine) and enzymes (e.g. tryptase) following stimulation either by IgE-mediated crosslinking with allergens or IgE-independent triggers (e.g. anaphylatoxins).
Mastocytosis: a clonal mast cell disorder, characterised by the proliferation, accumulation and activation of MCs in the skin and/or extracutaneous tissues.
Microdialysis: a research sampling technique that allows real-time measurements of extracellular concentrations of soluble endogenous and exogenous molecules from interstitial fluid through a semipermeable membrane [105].
Monocarboxylate acid transporters: members of the solute carrier (SLC) family of proteins
MRGPRX2 (MAS-related G-protein-coupled receptor member X2): a G-protein coupled receptor expressed in human MCs that is known as a potent activator of MCs [72].
Na^+^/K^+^-ATPase: a membrane ion pump system located in the sarcolemma, which requires a continuous source of ATP to function. For every ATP molecule, three NA^+^ ions are pumped out of the cell and two K^+^ ions are pumped into the cell [152].
Ovalbumin: a 45 kDa chicken-derived glycoprotein allergen, mostly present in egg white, which is commonly used for sensitization in animal models of anaphylaxis.
Prausnitz–Küstner Reaction: a test for type I hypersensitivity where a non-sensitized individual is injected intradermally with serum from an allergen-sensitized individual resulting in a subsequent wheal-and-flare reaction following administration of the causative allergen.
Wearable devices: miniaturized electronic device that can be easily donned on and off the body or incorporated into clothing or other body-worn accessories [168].
Warburg effect: metabolic phenomenon, originally described in cancer cells, where aerobic glycolysis is favoured over oxidative phosphorylation.

## Supplementary Information

Below is the link to the electronic supplementary material.

Supplementary file1 (PDF 478 KB)

## Abbreviations

AD: Atopic dermatitis
ATP: Adenosine triphosphate
CINDUs: Chronic inducible urticarias
CCL2: C-C motif chemokine ligand 2
COLD-CE: Comprehensive evaluation of cold urticaria and other cold-induced reactions, a study of the GA^2^LEN UCARE network
COVID-19: Coronavirus disease-2019
EAACI: European Academy of Allergy and Clinical Immunology
ED: Emergency department
EIA: Exercise-induced anaphylaxis
FDEIA: Food-dependent exercise-induced anaphylaxis
HDAC: Histone deacetylase
HIF-1α: Hypoxia-inducible factor 1-alpha
HR: Histamine release
ICU: Intensive care unit
IgE: Immunoglobulin E
*K*_m_: Michaelis constant
LAD2 cells: The Laboratory of Allergic Diseases mast cell line
LDH: Lactate dehydrogenase
MC: Mast cell
MCT1: Monocarboxylate transporter 1
MRGPRX2: Mas-related G protein-coupled receptor X2
mTOR: Mammalian target of rapamycin
NAD: Nicotinamide adenine dinucleotide
NADH: Nicotinamide adenine dinucleotide hydrogen
NRS: Numeric rating scale
ROC: Receiver operating characteristic
POEM: Patient-oriented eczema measure
TLR: Toll-like receptor
UCARE: GA^2^LEN network of urticaria centres of reference and excellence
VEGF: Vascular endothelial growth factor

## References

[1] Bartnikas LM, Sicherer SH. Fatal anaphylaxis: searching for lessons from tragedy. J Allergy Clin Immunol Pract. 2020;8(1):334–5. 31950906 10.1016/j.jaip.2019.11.005 PMC7461658

[2] Cardona V, Ansotegui IJ, Ebisawa M, et al. World Allergy Organization anaphylaxis guidance 2020. World Allergy Organ J. 2020;13(10): 100472. 33204386 10.1016/j.waojou.2020.100472 PMC7607509

[3] Shaker MS, Wallace DV, Golden DBK, et al. Anaphylaxis—a 2020 practice parameter update, systematic review, and Grading of Recommendations, Assessment, Development and Evaluation (GRADE) analysis. J Allergy Clin Immunol. 2020;145(4):1082–123. 32001253 10.1016/j.jaci.2020.01.017

[4] Turner PJ, Worm M, Ansotegui IJ, et al. Time to revisit the definition and clinical criteria for anaphylaxis? WAO Anaphylaxis Committee. World Allergy Organ J. 2019;12(10): 100066. 31719946 10.1016/j.waojou.2019.100066 PMC6838992

[5] Lee S, Hess EP, Lohse C, Gilani W, Chamberlain AM, Campbell RL. Trends, characteristics, and incidence of anaphylaxis in 2001–2010: a population-based study. J Allergy Clin Immunol. 2017;139(1):182–8. 27378753 10.1016/j.jaci.2016.04.029 PMC5182191

[6] Baseggio Conrado A, Ierodiakonou D, Gowland MH, Boyle RJ, Turner PJ. Food anaphylaxis in the United Kingdom: analysis of national data, 1998–2018. BMJ. 2021;372: n251. 33597169 10.1136/bmj.n251 PMC7885259

[7] Simons FE, Sampson HA. Anaphylaxis epidemic: fact or fiction? J Allergy Clin Immunol. 2008;122(6):1166–8. 19084110 10.1016/j.jaci.2008.10.019

[8] Ma L, Danoff TM, Borish L. Case fatality and population mortality associated with anaphylaxis in the United States. J Allergy Clin Immunol. 2014;133(4):1075–83. 24332862 10.1016/j.jaci.2013.10.029 PMC3972293

[9] Mullins RJ, Dear KB, Tang ML. Time trends in Australian hospital anaphylaxis admissions in 1998–1999 to 2011–2012. J Allergy Clin Immunol. 2015;136(2):367–75. 26187235 10.1016/j.jaci.2015.05.009

[10] Vadas P, Gold M, Perelman B, et al. Platelet-activating factor, PAF acetylhydrolase, and severe anaphylaxis. New Eng J Med. 2008;358(1):28–35. 18172172 10.1056/NEJMoa070030

[11] Layritz CM, Hagel AF, Graf V, Reiser C, et al. Histamine in atrial fibrillation (AF)—is there any connection? Results from an unselected population. Int J Cardiol. 2014;172(3):e432–3. 24476699 10.1016/j.ijcard.2013.12.185

[12] Pałgan K. Mast cells and basophils in IgE-independent anaphylaxis. Int J Mol Sci. 2023;24(16):12802. 37628983 10.3390/ijms241612802 PMC10454702

[13] Ono E, Taniguchi M, Mita H, Fukutomi Y, Higashi N, Miyazaki E, Kumamoto T, Akiyama K. Increased production of cysteinyl leukotrienes and prostaglandin D2 during human anaphylaxis. Clin Exp Allergy. 2009;39(1):72–80. 19128354 10.1111/j.1365-2222.2008.03104.x

[14] Kaplan AP. Preventing anaphylaxis fatalities: should we target bradykinin? J Allergy Clin Immunol. 2020;145(5):1365–6. 32035986 10.1016/j.jaci.2020.01.043

[15] Höfer V, Dölle-Bierke S, Francuzik W, et al. Fatal and near-fatal anaphylaxis: data from the European Anaphylaxis Registry and national health statistics. J Allergy Clin Immunol Pract. 2024;12(1):96-105.e8. 37816460 10.1016/j.jaip.2023.09.044

[16] Turner PJ, Gowland MH, Sharma V, et al. Increase in anaphylaxis-related hospitalizations but no increase in fatalities: an analysis of United Kingdom national anaphylaxis data, 1992–2012. J Allergy Clin Immunol. 2015;135(4):956–63. 25468198 10.1016/j.jaci.2014.10.021 PMC4382330

[17] Schwartz LB, Delgado L, Craig T, et al. Exercise-induced hypersensitivity syndromes in recreational and competitive athletes: a PRACTALL consensus report (what the general practitioner should know about sports and allergy). Allergy. 2008;63(8):953–61. 18691297 10.1111/j.1398-9995.2008.01802.x

[18] Bizjak M, Košnik M, Dinevski D, et al. Risk factors for systemic reactions in typical cold urticaria: results from COLD-CE study. Allergy. 2022;77(7):2185–99. 34862605 10.1111/all.15194

[19] Beck SC, Wilding T, Buka RJ, Baretto RL, Huissoon AP, Krishna MT. Biomarkers in human anaphylaxis: a critical appraisal of current evidence and perspectives. Front Immunol. 2019;10:494. 31024519 10.3389/fimmu.2019.00494 PMC6459955

[20] Dribin TE, Schnadower D, Wang J, et al. Anaphylaxis knowledge gaps and future research priorities: a consensus report. J Allergy Clin Immunol. 2022;149(3):999–1009. 34390722 10.1016/j.jaci.2021.07.035 PMC8837706

[21] Dua S, Ruiz-Garcia M, Bond S, Durham SR, Kimber I, Mills C, Roberts G, Skypala I, Wason J, Ewan P, Boyle R, Clark A. Effect of sleep deprivation and exercise on reaction threshold in adults with peanut allergy: a randomized controlled study. J Allergy Clin Immunol. 2019;144(6):1584-1594.e2. 31319102 10.1016/j.jaci.2019.06.038 PMC6904229

[22] Kulthanan K, Ungprasert P, Jirapongsananuruk O, et al. Food-dependent exercise-induced wheals, angioedema, and anaphylaxis: a systematic review. J Allergy Clin Immunol Pract. 2022;10(9):2280–96. 35752432 10.1016/j.jaip.2022.06.008

[23] Jimenez-Rodriguez TW, Garcia-Neuer M, Alenazy LA, Castells M. Anaphylaxis in the 21st century: phenotypes, endotypes and biomarkers. J Asthma Allergy. 2018;11:121–42. 29950872 10.2147/JAA.S159411 PMC6016596

[24] Giannetti MP, Weller E, Bormans C, Novak P, Hamilton MJ, Castells M. Hereditary alpha-tryptasemia in 101 patients with mast cell activation-related symptomatology including anaphylaxis. Ann Allergy Asthma Immunol. 2021;126(6):655–60. 33465452 10.1016/j.anai.2021.01.016

[25] Polivka L, Madrange M, Bulai-Livideanu C, CEREMAST Network, et al. Pathophysiologic implications of elevated prevalence of hereditary alpha-tryptasemia in all mastocytosis subtypes. J Allergy Clin Immunol. 2024;153(1):349.e4-353.e4. 37633651 10.1016/j.jaci.2023.08.015

[26] Mateja A, Wang Q, Chovanec J, Kim J, Wilson KJ, Schwartz LB, Glover SC, Carter MC, Metcalfe DD, Brittain E, Lyons JJ. Defining baseline variability of serum tryptase levels improves accuracy in identifying anaphylaxis. J Allergy Clin Immunol. 2022;149(3):1010-1017.e10. 34425177 10.1016/j.jaci.2021.08.007 PMC9126046

[27] Galvan-Blasco P, Gil-Serrano J, Sala-Cunill A. New biomarkers in anaphylaxis (beyond tryptase). Curr Treat Options Allergy. 2022;9(4):303–22. 36467524 10.1007/s40521-022-00326-1 PMC9702867

[28] Castells M. Diagnosis and management of anaphylaxis in precision medicine. J Allergy Clin Immunol. 2017;140(2):321–33. 28780940 10.1016/j.jaci.2017.06.012

[29] Rossi CM, Lenti MV, Di Sabatino A. Adult anaphylaxis: a state-of-the-art review. Eur J Intern Med. 2022;100:5–12. 35264295 10.1016/j.ejim.2022.03.003

[30] Castells MC, Li TJ. Anaphylaxis: parts unknown. J Allergy Clin Immunol. 2020;8(4):1216–8. 10.1016/j.jaip.2020.01.017 32276690

[31] Guerci P, Tacquard C, Chenard L, et al. Epidemiology and outcome of patients admitted to intensive care after anaphylaxis in France: a retrospective multicentre study. Br J Anesthesia. 2020;125(6):1025–33. 10.1016/j.bja.2020.08.024 32928517

[32] Brooks GA, Osmond AD, Arevalo JA, et al. Lactate as a major myokine and exerkine. Nat Rev Endocrinol. 2022;18(11):712. 35915256 10.1038/s41574-022-00724-0

[33] Zunz E, La Barre J. A propos des variations du sucre libre, du sucre protéidique et de l’acide lactique lors du choc anaphylactique chez le cobaye. Compt Rend Soc de Biol. 1924;III:121–5.

[34] Meyerhof O. Über die Rolle der Milchsäure in der Energetik des Muskels. Naturwissenschaften. 1920;8:696–704.

[35] Hill AV, Long CNH, Lupton H. Muscular exercise, lactic acid, and the supply and utilisation of oxygen—parts ӏ–ӏӏӏ. R Soc Lond. 1924;96:438–75.

[36] Warburg O, Posener K, Negelein E. Über den Stoffwechsel der Tumoren. Biochem Z. 1924;152:319–44.

[37] Warburg O. The metabolism of carcinoma cells. J Cancer Res. 1925;9:148–63.

[38] DeBerardinis RJ, Chandel NS. We need to talk about the Warburg effect. Nat Met. 2020;2(2):127–9. 10.1038/s42255-020-0172-2 32694689

[39] Brooks GA. The science and translation of lactate shuttle theory. Cel Metab. 2018;27(4):757–85. 10.1016/j.cmet.2018.03.008 29617642

[40] Brooks GA. The tortuous path of lactate shuttle discovery: from cinders and boards to the lab and ICU. J Sport Health Sci. 2020;9(5):446–60. 32444344 10.1016/j.jshs.2020.02.006 PMC7498672

[41] Certo M, Llibre A, Lee W, Mauro C. Understanding lactate sensing and signaling. Trends Endocrinol Metab. 2022;33(10):722–35. 35999109 10.1016/j.tem.2022.07.004

[42] Wu Y, Ma W, Liu W, Zhang S. Lactate: a pearl dropped in the ocean—an overlooked signal molecule in physiology and pathology. Cell Biol Int. 2023;47(2):295–307. 36511218 10.1002/cbin.11975

[43] Ippolito L, Sonveaux P, Chiarugi P. Unconventional roles of lactate along the tumor and immune landscape. Trends Endocrinol Metab. 2022;33(4):231–5. 35168874 10.1016/j.tem.2022.01.005

[44] Castells M. Mast cell disorders. A framework of allergy and hematology symptoms leading to personalized treatments. Ann Allergy Asthma Immunol. 2021;127(4):403–4. 34593102 10.1016/j.anai.2021.07.025

[45] Levy B. Lactate and shock state: the metabolic view. Curr Opin Crit Care. 2006;12(4):315–21. 16810041 10.1097/01.ccx.0000235208.77450.15

[46] Vincent JL, De Backer D. Circulatory shock. N Engl J Med. 2013;369(18):1726–34. 24171518 10.1056/NEJMra1208943

[47] Perales-Chorda C, Obeso D, Twomey L, et al. Characterization of anaphylaxis reveals different metabolic changes depending on severity and triggers. Clin Exp Allergy. 2021;51(10):1295–309. 34310748 10.1111/cea.13991

[48] Ostroukhova M, Goplen N, Karim MZ, Michalec L, Guo L, Liang Q, Alam R. The role of low-level lactate production in airway inflammation in asthma. Am J Physiol Lung Cell Mol Physiol. 2012;302(3):L300–7. 22080752 10.1152/ajplung.00221.2011 PMC3289274

[49] Lee SY, Park YM, Yoo HJ, Hong SJ. Metabolomic pathways in food allergy. Pediatr Allergy Immunol. 2024;35(5): e14133. 38727629 10.1111/pai.14133

[50] Crestani E, Harb H, Charbonnier LM, Leirer J, Motsinger-Reif A, Rachid R, Phipatanakul W, Kaddurah-Daouk R, Chatila TA. Untargeted metabolomic profiling identifies disease-specific signatures in food allergy and asthma. J Allergy Clin Immunol. 2020;145(3):897–906. 31669435 10.1016/j.jaci.2019.10.014 PMC7062570

[51] Metcalfe DD, Peavy RD, Gilfillan AM. Mechanisms of mast cell signaling in anaphylaxis. J Allergy Clin Immunol. 2009;124(4):639–46. 19815110 10.1016/j.jaci.2009.08.035 PMC2788154

[52] Navinés-Ferrer A, Serrano-Candelas E, Lafuente A, Muñoz-Cano R, Martín M, Gastaminza G. MRGPRX2-mediated mast cell response to drugs used in perioperative procedures and anaesthesia. Sci Rep. 2018;8(1):11628. 30072729 10.1038/s41598-018-29965-8 PMC6072780

[53] Bawazir M, Sutradhar S, Roy S, Ali H. MRGPRX2 facilitates IgE-mediated systemic anaphylaxis in a newly established knock-in mouse model. J Allergy Clin Immunol. 2025;155(3):974-987.e1. 39581296 10.1016/j.jaci.2024.11.021

[54] Schwartz LB, Metcalfe DD, Miller JS, Earl H, Sullivan T. Tryptase levels as an indicator of mast-cell activation in systemic anaphylaxis and mastocytosis. N Engl J Med. 1987;316(26):1622–6. 3295549 10.1056/NEJM198706253162603

[55] Sheffer AL, Soter NA, McFadden ER Jr, Austen KF. Exercise-induced anaphylaxis: a distinct form of physical allergy. J Allergy Clin Immunol. 1983;71(3):311–6. 6826991 10.1016/0091-6749(83)90085-4

[56] Chakravarty N, Sorensen HJ. Stimulation of glucose metabolism in rat mast cells by antigen, dextran and compound 48–80, used as histamine releasing agents. Acta Physiol Scand. 1974;91(3):339–53. 4135948 10.1111/j.1748-1716.1974.tb05689.x

[57] Johansen T. Adenosine triphosphate levels during histamine release induced by compound 48/80 in rat mast cells in vitro. Life Sci. 1980;26(1):61–9. 6153745 10.1016/0024-3205(79)90189-9

[58] Johansen T. Further observations on the utilization of adenosine triphosphate in rat mast cells during histamine release induced by the ionophore A23187. Br J Pharmacol. 1980;69(4):657–62. 6159939 10.1111/j.1476-5381.1980.tb07918.x PMC2044306

[59] Yoshizaki K, Arizono N. Simultaneous detection of histamine release and lactate production in rat mast cells induced by compound 48/80 using 1H NMR. Exp Cell Res. 1991;193(2):279–82. 1706276 10.1016/0014-4827(91)90097-e

[60] Johansen T. Utilization of adenosine triphosphate in rat mast cells during and after secretion of histamine in response to compound 48/80. Acta Pharmacol Toxicol (Copenh). 1983;53(3):245–9. 6195889 10.1111/j.1600-0773.1983.tb01132.x

[61] Yoshizaki K, Arizono N, Hayano T, Watari H. Allergen-induced histamine secretion associated with lactate production in mast cells detected by 1H NMR. Magn Reson Med. 1993;29(6):732–6. 7688844 10.1002/mrm.1910290604

[62] Hosoda S, Glick D. Biosynthesis of 5-hydroxytryptophan and 5-hydroxytryptamine from tryptophan by neoplastic mouse mast cells. Biochim Biophys Acta. 1965;111(1):67–78. 5295589 10.1016/0304-4165(65)90473-3

[63] Diamant B, Glick D. Certain dehydrogenase activities and dry weight of normal and malignant mast cells. J Histochem Cytochem. 1967;15(11):695–701. 4871625 10.1177/15.11.695

[64] Diamant B, Peterson C. The metabolism of monosaccharides in isolated rat mast cells and its influence on histamine release induced by adenosine-5′-triphosphate. Acta Physiol Scand. 1971;83(3):324–34. 4109132 10.1111/j.1748-1716.1971.tb05085.x

[65] Chakravarty N, Sorensen HJ. Stimulation of glucose metabolism in rat mast cells by antigen, dextran and compound 48–80, used as histamine releasing agents. Acta Physiol Scand. 1974;91:339–53. 4135948 10.1111/j.1748-1716.1974.tb05689.x

[66] Johansen T. Adenosine triphosphate levels during anaphylactic histamine release in rat mast cells in vitro. Effects of glycolytic and respiratory inhibitors. Eur J Pharmacol. 1979;58(2):107–15. 91528 10.1016/0014-2999(79)90001-3

[67] Fewtrell C, Lagunoff D, Metzger H. Secretion from rat basophilic leukaemia cells induced by calcium ionophores. Effect of pH and metabolic inhibition. Biochim Biophys Acta. 1981;644(2):363–8. 6789877 10.1016/0005-2736(81)90394-1

[68] Abebayehu D, Spence AJ, Qayum AA, et al. Lactic acid suppresses IL-33-mediated mast cell inflammatory responses via hypoxia-inducible factor-1α-dependent miR-155 suppression. J Immunol. 2016;197(7):2909–17. 27559047 10.4049/jimmunol.1600651 PMC5026940

[69] Abebayehu D, Spence AJ, Caslin H, et al. Lactic acid suppresses IgE-mediated mast cell function in vitro and in vivo. Cell Immunol. 2019;341: 103918. 31030957 10.1016/j.cellimm.2019.04.006 PMC6579658

[70] Caslin HL, Abebayehu D, Abdul Qayum A, et al. Lactic acid inhibits lipopolysaccharide-induced mast cell function by limiting glycolysis and ATP availability. J Immunol. 2019;203(2):453–64. 31160535 10.4049/jimmunol.1801005 PMC6734564

[71] Mendoza RP, Anderson CC, Fudge DH, Roede JR, Brown JM. Metabolic consequences of IgE- and non-IgE-mediated mast cell degranulation. J Immunol. 2021;207:2637–48. 34732470 10.4049/jimmunol.2001278 PMC8612977

[72] Syed M, Kammala AK, Callahan B, Oskeritzian CA, Subramanian H. Lactic acid suppresses MRGPRX2 mediated mast cell responses. Cell Immunol. 2021;368: 104422. 34399172 10.1016/j.cellimm.2021.104422 PMC8428143

[73] Chelombitko MA, Chernyak BV, Fedorov AV, Zinovkin RA, Razin E, Paruchuru LB. The role played by mitochondria in FcεRI-dependent mast cell activation. Front Immunol. 2020;11: 584210. 33178217 10.3389/fimmu.2020.584210 PMC7596649

[74] Mendoza RP, Fudge DH, Brown JM. Cellular energetics of mast cell development and activation. Cells. 2021;10(3):524. 33801300 10.3390/cells10030524 PMC7999080

[75] Sumbayev VV, Yasinska I, Oniku AE, Streatfield CL, Gibbs BF. Involvement of hypoxia-inducible factor-1 in the inflammatory responses of human LAD2 mast cells and basophils. PLoS ONE. 2012;7(3): e34259. 22470546 10.1371/journal.pone.0034259 PMC3314605

[76] Gibbs BF, Yasinska IM, Pchejetski D, Wyszynski RW, Sumbayev VV. Differential control of hypoxia-inducible factor 1 activity during pro-inflammatory reactions of human haematopoietic cells of myeloid lineage. Int J Biochem Cell Biol. 2012;44(11):1739–49. 22728164 10.1016/j.biocel.2012.06.019

[77] Iskarpatyoti JA, Shi J, Abraham MA, Rathore APS, Miao Y, Abraham SN. Mast cell regranulation requires a metabolic switch involving mTORC1 and a glucose-6-phosphate transporter. Cell Rep. 2022;40(13): 111346. 36170813 10.1016/j.celrep.2022.111346 PMC11218746

[78] Goretzki A, Lin YJ, Schülke S. Immune metabolism in allergies, does it matter? A review of immune metabolic basics and adaptations associated with the activation of innate immune cells in allergy. Allergy. 2021;76(11):3314–31. 33811351 10.1111/all.14843

[79] Sumbayev VV, Nicholas SA, Streatfield CL, Gibbs BF. Involvement of hypoxia-inducible factor-1 HiF(1alpha) in IgE-mediated primary human basophil responses. Eur J Immunol. 2009;39(12):3511–9. 19768695 10.1002/eji.200939370

[80] Johansen T, Chakravarty N. Adenosine triphosphate content of mast cells in relation to histamine release induced by anaphylactic reaction. Int Arch Allergy App Immunol. 1975;49(1–2):208. 10.1159/000231395 49314

[81] Rabinowitz JD, Enerbäck S. Lactate: the ugly duckling of energy metabolism. Nat Metab. 2020;2(7):566–71. 32694798 10.1038/s42255-020-0243-4 PMC7983055

[82] Nasu Y, Murphy-Royal C, Wen Y, et al. A genetically encoded fluorescent biosensor for extracellular l-lactate. Nat Commun. 2021;12(1):7058. 34873165 10.1038/s41467-021-27332-2 PMC8648760

[83] Harada K, Chihara T, Hayasaka Y, et al. Green fluorescent protein-based lactate and pyruvate indicators suitable for biochemical assays and live cell imaging. Sci Rep. 2020;10(1):19562. 33177605 10.1038/s41598-020-76440-4 PMC7659002

[84] Amantonico A, Urban PL, Zenobi R. Analytical techniques for single-cell metabolomics: state of the art and trends. Anal Bioanal Chem. 2010;398(6):2493–504. 20544183 10.1007/s00216-010-3850-1

[85] Caslin HL, Abebayehu D, Pinette JA, Ryan JJ. Lactate is a metabolic mediator that shapes immune cell fate and function. Front Physiol. 2021;12: 688485. 34733170 10.3389/fphys.2021.688485 PMC8558259

[86] Bosshart PD, Charles RP, Garibsingh RA, Schlessinger A, Fotiadis D. SLC16 family: from atomic structure to human disease. Trends Biochem Sci. 2021;46(1):28–40. 32828650 10.1016/j.tibs.2020.07.005

[87] Ghannadan M, Baghestanian M, Wimazal F, et al. Phenotypic characterization of human skin mast cells by combined staining with toluidine blue and CD antibodies. J Investig Dermatol. 1998;111(4):689–95. 9764855 10.1046/j.1523-1747.1998.00359.x

[88] Robergs RA, McNulty CR, Minett GM, Holland J, Trajano G. Lactate, not lactic acid, is produced by cellular cytosolic energy catabolism. Physiology (Bethesda). 2018;33(1):10–2. 29212886 10.1152/physiol.00033.2017

[89] Reber LL, Hernandez JD, Galli SJ. The pathophysiology of anaphylaxis. J Allergy Clin Immunol. 2017;140(2):335–48. 28780941 10.1016/j.jaci.2017.06.003 PMC5657389

[90] Ziogas A, Sajib MS, Lim JH, et al. Glycolysis is integral to histamine-induced endothelial hyperpermeability. FASEB J. 2021;35(3): e21425. 33566443 10.1096/fj.202001634R PMC7909462

[91] Romero SA, McCord JL, Ely MR, et al. Mast cell degranulation and *de novo* histamine formation contribute to sustained postexercise vasodilation in humans. J Appl Physiol. 2017;122(3):603–10. 27562843 10.1152/japplphysiol.00633.2016 PMC5401950

[92] Van der Stede T, Blancquaert L, Stassen F, Everaert I, et al. Histamine H_1_ and H_2_ receptors are essential transducers of the integrative exercise training response in humans. Sci Adv. 2021;7(16):eabf2856. 33853781 10.1126/sciadv.abf2856 PMC8046361

[93] Parsons IT, Stacey MJ, Faconti L, et al. Histamine, mast cell tryptase and post-exercise hypotension in healthy and collapsed marathon runners. Eur J Appl Physiol. 2021;121(5):1451–9. 33629149 10.1007/s00421-021-04645-0 PMC8373737

[94] San-Millán I, Brooks GA. Re-examining cancer metabolism: lactate production for cancerogenesis could be the purpose and explanation of the Warburg Effect. Carcinogenesis. 2017;38(2):119–33. 27993896 10.1093/carcin/bgw127 PMC5862360

[95] Portier P, Richet C. De l’action anaphylactique de certains venins. C R Seances Soc Biol. 1902;54:170–2.

[96] Gouel-Chéron A, Dejoux A, Lamanna E, Bruhns P. Animal models of IgE anaphylaxis. Biology (Basel). 2023;12(7):931. 37508362 10.3390/biology12070931 PMC10376466

[97] Paul P, Holmes WL. Free fatty acid metabolism during stress: exercise, acute cold exposure, and anaphylactic shock. Lipids. 1973;8(3):142–50. 4692881 10.1007/BF02531811

[98] Fredholm BB, Strandberg K. Some metabolic consequences of the anaphylactic reaction in the rabbit. Acta Physio Scand. 1975;93(1):52–8. 10.1111/j.1748-1716.1975.tb05789.x 1155131

[99] Dewachter P, Jouan-Hureaux V, Franck P, et al. Anaphylactic shock: a form of distributive shock without inhibition of oxygen consumption. Anesthesiology. 2005;103(1):40–9. 15983455 10.1097/00000542-200507000-00010

[100] Tajima K, Zheng F, Collange O, et al. Time to achieve target mean arterial pressure during resuscitation from experimental anaphylactic shock in an animal model. A comparison of adrenaline alone or in combination with different volume expanders. Anaesth Intensive Care. 2013;41(6):765–73. 24180718 10.1177/0310057X1304100612

[101] Platts-Mills TAE, Eid RC, Keshavarz B. Of mice and men, MMXXI: anaphylaxis. J Allergy Clin Immunol. 2022;149(1):58–9. 34599978 10.1016/j.jaci.2021.08.029

[102] Burton OT, Stranks AJ, Tamayo JM, Koleoglou KJ, Schwartz LB, Oettgen HC. A humanized mouse model of anaphylactic peanut allergy. J Allergy Clin Immunol. 2017;139(1):314-322.e9. 27417025 10.1016/j.jaci.2016.04.034 PMC5145786

[103] Kanagaratham C, Sallis BF, Fiebiger E. Experimental models for studying food allergy. Cell Mol Gastroenterol Hepatol. 2018;6(3):356-369.e1. 30182049 10.1016/j.jcmgh.2018.05.010 PMC6121159

[104] Arul Arasan TS, Jorgensen R, Van Antwerp C, Ng PKW, Gangur V. Advances in mechanisms of anaphylaxis in wheat allergy: utility of rodent models. Foods. 2025;14(5):883. 40077585 10.3390/foods14050883 PMC11899146

[105] Ungerstedt U. Microdialysis—a new technique for monitoring local tissue events in the clinic. Acta Anaesthesiol Scand Suppl. 1997;110:123. 9248559 10.1111/j.1399-6576.1997.tb05527.x

[106] Baumann KY, Church MK, Clough GF, et al. Skin microdialysis: methods, applications and future opportunities—an EAACI position paper. Clin Trans Allergy. 2019;9:24. 10.1186/s13601-019-0262-y PMC6456961 31007896

[107] Petersen LJ. Interstitial lactate levels in human skin at rest and during an oral glucose load: a microdialysis study. Clin Physiol. 1999;19(3):246–50. 10361615 10.1046/j.1365-2281.1999.00174.x

[108] Rosdahl H, Ungerstedt U, Jorfeldt L, Henriksson J. Interstitial glucose and lactate balance in human skeletal muscle and adipose tissue studied by microdialysis. J Physiol. 1993;471:637–57. 8120827 10.1113/jphysiol.1993.sp019920 PMC1143981

[109] Krogstad AL, Jansson PA, Gisslén P, Lönnroth P. Microdialysis methodology for the measurement of dermal interstitial fluid in humans. Br J Dermatol. 1996;134(6):1005–12. 8763416

[110] Jansson PA, Krogstad AL, Lönnroth P. Microdialysis measurements in skin: evidence for significant lactate release in healthy humans. Am J Physiol. 1996;271(1pt1):E138–42. 8760091 10.1152/ajpendo.1996.271.1.E138

[111] Decker RH. Nature and regulation of energy metabolism in the epidermis. J Investig Dermatol. 1971;57(6):351–63. 5001831 10.1111/1523-1747.ep12292707

[112] Johnson JA, Fusaro RM. The role of the skin in carbohydrate metabolism. Adv Metab Disord. 1972;60:1–55. 4581900 10.1016/b978-0-12-027306-5.50006-1

[113] Oharazawa A, Maimaituxun G, Watanabe K, Nishiyasu T, Fujii N. Metabolome analyses of skin dialysate: insights into skin interstitial fluid biomarkers. J Dermatol Sci. 2024;114(3):141–7. 38740531 10.1016/j.jdermsci.2024.04.001

[114] MacLean DA, Bangsbo J, Saltin B. Muscle interstitial glucose and lactate levels during dynamic exercise in humans determined by microdialysis. J Appl Physiol. 1999;87(4):1483–90. 10517782 10.1152/jappl.1999.87.4.1483

[115] Shen Y, Liu C, He H, et al. Recent advances in wearable biosensors for non-invasive detection of human lactate. Biosensors (Basel). 2022;12(12):1164. 36551131 10.3390/bios12121164 PMC9776101

[116] Lewis J, Lieberman P, Treadwell G, Erffmeyer J. Exercise-induced urticaria, angioedema, and anaphylactoid episodes. J Allergy Clin Immunol. 1981;68(6):432–7. 7310010 10.1016/0091-6749(81)90197-4

[117] Castells MC, Horan RF, Sheffer AL. Exercise-induced anaphylaxis. Clin Rev Allergy Immunol. 1999;17(4):413–24. 10829811 10.1007/BF02737646

[118] Nalbandian M, Takeda M. Lactate as a signaling molecule that regulates exercise-induced adaptations. Biology (Basel). 2016;5(4):38. 27740597 10.3390/biology5040038 PMC5192418

[119] Brooks GA. Intra- and extra-cellular lactate shuttles. Med Sci Sports Exerc. 2000;32(4):790–9. 10776898 10.1097/00005768-200004000-00011

[120] Mayes R, Hardman AE, Williams C. The influence of training on endurance and blood lactate concentration during submaximal exercise. Br J Sports Med. 1987;21(3):119–24. 3676637 10.1136/bjsm.21.3.119 PMC1478437

[121] Benítez-Muñoz JA, Cupeiro R, Rubio-Arias JÁ, Amigo T, González-Lamuño D. Exercise influence on monocarboxylate transporter 1 (MCT1) and 4 (MCT4) in the skeletal muscle: a systematic review. Acta Physiol. 2024;240(3): e14083. 10.1111/apha.14083 38240467

[122] Pillon NJ, Gabriel BM, Dollet L, et al. Transcriptomic profiling of skeletal muscle adaptations to exercise and inactivity. Nat Commun. 2020;11(1):470. 31980607 10.1038/s41467-019-13869-w PMC6981202

[123] Romero SA, Hocker AD, Mangum JEL, et al. Evidence of a broad histamine footprint on the human exercise transcriptome. J Physiol. 2016;594(17):5009–23. 27061420 10.1113/JP272177 PMC5009782

[124] Luttrell MJ, Halliwill JR. The intriguing role of histamine in exercise responses. Exerc Sport Sci Rev. 2017;45(1):16–23. 27741023 10.1249/JES.0000000000000093 PMC5161583

[125] Dunér H, Pernow B. Histamine and leukocytes in blood during muscular work in man. Scand J Clin Lab Investig. 1958;10(4):394–6. 13615245 10.3109/00365515809051243

[126] Yan H, Behun MA, Cook MD, et al. Differential post-exercise blood pressure responses between Blacks and Caucasians. PLoS ONE. 2016;11(4): e0153445. 27074034 10.1371/journal.pone.0153445 PMC4830622

[127] Worm M, Francuzik W, Renaudin JM, et al. Factors increasing the risk for a severe reaction in anaphylaxis: an analysis of data from the European Anaphylaxis Registry. Allergy. 2018;73(6):1322–30. 29318637 10.1111/all.13380

[128] Magerl M, Altrichter S, Borzova E, et al. The definition, diagnostic testing, and management if chronic inducible urticarias—the EAACI/GA^2^LEN/EDF/UNEV consensus recommendations 2016 update and revision. Allergy. 2016;71(6):780–802. 26991006 10.1111/all.12884

[129] Christensen MJ, Eller E, Mortz CG, Brockow K, Bindslev-Jensen C. Exercise lowers threshold and increases severity, but wheat-dependent, exercise-induced anaphylaxis can be elicited at rest. J Allergy Clin Immunol Pract. 2018;6(2):514–20. 29524997 10.1016/j.jaip.2017.12.023

[130] Ansley L, Bonini M, Delgado L, et al. Pathophysiological mechanisms of exercise-induced anaphylaxis: an EAACI position statement. Allergy. 2015;70(10):1212–21. 26100553 10.1111/all.12677

[131] Mikhailov P, Berova N, Andreev VC. Physical urticaria and sport. Cutis. 1977;20(3):389–90. 891255

[132] Sheffer AL, Austin KF. Exercise-induced anaphylaxis. J Allergy Clin Immunol. 1980;66(2):106–11. 7400473 10.1016/0091-6749(80)90056-1

[133] Sheffer AL, Tong AK, Murphy GF, Lewis RA, McFadden ER Jr, Austen KF. Exercise-induced anaphylaxis: a serious form of physical allergy associated with mast cell degranulation. J Allergy Clin Immunol. 1985;75(4):479–84. 3980883 10.1016/s0091-6749(85)80021-x

[134] Sheffer AL, Austen KF. Exercise-induced anaphylaxis. J Allergy Clin Immunol. 1984;73(5pt2):699–703. 6715733 10.1016/0091-6749(84)90309-9

[135] Casale TB, Keahey TM, Kaliner M. Exercise-induced anaphylactic syndromes. Insights into diagnostic and pathophysiologic features. JAMA. 1986;255(15):2049–53. 3514973

[136] Tse KS, et al. A study of exercise-induced urticaria and angioedema. J Allergy Clin Immunol. 1980;65:228.

[137] Schwartz HJ. Elevated serum tryptase in exercise-induced anaphylaxis. J Allergy Clin Immunol. 1995;95(4):917–9. 7722176 10.1016/s0091-6749(95)70139-7

[138] Stephansson E, Koskimies S, Lokki ML. Exercise-induced urticaria and anaphylaxis. Acta Derm Venereol. 1991;71(2):138–42. 1675522

[139] Silvers WS. Exercise-induced allergies: the role of histamine release. Ann Allergy. 1992;68(1):58–63. 1371041

[140] Chow LS, Gerszten RE, Taylor JM, et al. Exerkines in health, resilience and disease. Nat Rev Endocrinol. 2022;18(5):273–89. 35304603 10.1038/s41574-022-00641-2 PMC9554896

[141] Maulitz RM, Pratt DS, Schocket AL. Exercise-induced anaphylactic reaction to shellfish. J Allergy Clin Immunol. 1979;63(6):433–4. 447945 10.1016/0091-6749(79)90218-5

[142] Adams BB. Exercise-induced anaphylaxis in a marathon runner. Int J Dermatol. 2002;41(7):394–6. 12121550 10.1046/j.1365-4362.2002.01454_2.x

[143] Brooks GA, Arevalo JA, Osmond AD, Leija RG, Curl CC, Tovar AP. Lactate in contemporary biology: a phoenix risen. J Physiol. 2022;600(5):1229–51. 33566386 10.1113/JP280955 PMC9188361

[144] Herlitz G. Exertion urticaria and lactic acid. Acta Allergol. 1949;2:44–50.

[145] Gülen T, Akin C. Anaphylaxis and mast cell disorder. Immunol Allergy Clin N Am. 2022;42(1):45–63. 10.1016/j.iac.2021.09.007 34823750

[146] Gülen T, Hägglund H, Dahlén B, Nilsson G. High prevalence of anaphylaxis in patients with systemic mastocytosis—a single-centre experience. Clin Exp Allergy. 2013;44(1):121–9. 10.1111/cea.12225 24164252

[147] Boehm T, Ristl R, Joseph S, Petroczi K, Klavins K, Valent P, Jilma B. Metabolome and lipidome derangements during a severe mast cell activation event in a patient with indolent systemic mastocytosis. J Allergy Clin Immunol. 2021;148(6):1533–44. 33864889 10.1016/j.jaci.2021.03.043

[148] Mungan I, Dicle CL, Ademoglu D, et al. The sequential evaluation of static and kinetic lactate levels as a predictor of mortality in patients with septic shock in the intensive care unit. J Crit Intensive Care. 2024;15(2):63–70.

[149] Fuernau G, Desch S, de Waha-Thiele S, Eitel I, Neumann FJ, Hennersdorf M, Felix SB, Fach A, Böhm M, Pöss J, Jung C, Ouarrak T, Schneider S, Werdan K, Zeymer U, Thiele H. Arterial lactate in cardiogenic shock: prognostic value of clearance versus single values. JACC Cardiovasc Interv. 2020;13(19):2208–16. 33032708 10.1016/j.jcin.2020.06.037

[150] Obeso D, Mera-Berriatua L, Rodríguez-Coira J, et al. Multi-omics analysis points to altered platelet functions in severe food-associated respiratory allergy. Allergy. 2018;73(11):2137–49. 30028518 10.1111/all.13563

[151] Mazzeo RS, Marshall P. Influence of plasma catecholamines on the lactate threshold during graded exercise. J Appl Physiol. 1989;67(4):1319–22. 2793730 10.1152/jappl.1989.67.4.1319

[152] Levy B, Gibot S, Franck P, Cravoisy A, Bollaert PE. Relation between muscle Na^+^K^+^ ATPase activity and raised lactate concentrations in septic shock: a prospective study. Lancet. 2005;365(9462):871–5. 15752531 10.1016/S0140-6736(05)71045-X

[153] Hanashiro PK, Weil MH. Anaphylactic shock in man. Report of two cases with detailed hemodynamic and metabolic studies. Arch Intern Med. 1967;119(2):129–40. 6017121 10.1001/archinte.119.2.129

[154] Oh HS, Chung CR, Park CM, Suh GY, Ko RE. Epinephrine-induced lactic acidosis during the management of anaphylactic shock: a case report. Clin Exp Emerg Med. 2024. 10.15441/ceem.24.239. 38778486 10.15441/ceem.24.239 PMC12861706

[155] Patel N, Chong KW, Yip AYG, Ierodiakonou D, Bartra J, Boyle RJ, Turner PJ. Use of multiple epinephrine doses in anaphylaxis: a systematic review and meta-analysis. J Allergy Clin Immunol. 2021;148(5):1307–15. 33862009 10.1016/j.jaci.2021.03.042 PMC8588837

[156] Turner PJ, Arasi S, Ballmer-Weber B, et al. Risk factors for severe reactions in food allergy: rapid evidence review with meta-analysis. Allergy. 2022;77(9):2634–52. 35441718 10.1111/all.15318 PMC9544052

[157] Langan SP, Navarro JS. How high can the lactate phoenix rise? J Physiol. 2022;600(11):2813–4. 35538348 10.1113/JP283089

[158] Goodwin ML, Harris JE, Hernández A, Gladden LB. Blood lactate measurements and analysis during exercise: a guide for clinicians. J Diabetes Sci Technol. 2007;1(4):558–69. 19885119 10.1177/193229680700100414 PMC2769631

[159] Kirschenbaum LA, Astiz ME, Rackow EC. Interpretation of blood lactate concentrations in patients with sepsis. Lancet. 1998;352(9132):921–2. 9752810 10.1016/S0140-6736(05)61507-3

[160] Smith ZR, Horng M, Rech MA. Medication-induced hyperlactatemia and lactic acidosis: a systematic review of the literature. Pharmacotherapy. 2019;39(9):946–63. 31361914 10.1002/phar.2316

[161] Wardi G, Brice J, Correia M, Liu D, Self M, Tainter C. Demystifying lactate in the emergency department. Ann Emerg Med. 2020;75:287–98. 31474479 10.1016/j.annemergmed.2019.06.027

[162] Biomarkers Definitions Working Group. Biomarkers and surrogate endpoints: preferred definitions and conceptual framework. Clin Pharmacol Ther. 2001;69(3):89–95. 11240971 10.1067/mcp.2001.113989

[163] Strimbu K, Tavel JA. What are biomarkers? Curr Opin HIV AIDS. 2010;5(6):463–6. 20978388 10.1097/COH.0b013e32833ed177 PMC3078627

[164] Nakahara T, Onozuka D, Nunomura S, et al. The ability of biomarkers to assess the severity of atopic dermatitis. J Allergy Clin Immunol Glob. 2024;3(1): 100175. 37915726 10.1016/j.jacig.2023.100175 PMC10616407

[165] Wollenberg A, Beck LA, Blauvelt A. Laboratory safety of dupilumab in moderate-to-severe atopic dermatitis: results from three phase III trials (LIBERTY AD SOLO 1, LIBERTY AD SOLO 2, LIBERTY AD CHRONOS). Br J Dermatol. 2020;182(5):1120–35. 31407311 10.1111/bjd.18434 PMC7317598

[166] Roncoroni AJ, Adrougué HJ, De Obrutsky CW, Marchisio ML, Herrera MR. Metabolic acidosis in status asthmaticus. Respiration. 1976;33(2):85–94. 778959 10.1159/000193721

[167] Ruman-Colombier M, Rochat Guignard I, Di Paolo ER, Gehri M, Pauchard JY. Prevalence and risk factors of lactic acidosis in children with acute moderate and severe asthma, a prospective observational study. Eur J Pediatr. 2021;180(4):1125–31. 33089387 10.1007/s00431-020-03834-x PMC7940309

[168] Smuck M, Odonkor CA, Wilt JK, Schmidt N, Swiernik MA. The emerging clinical role of wearables: factors for successful implementation in healthcare. NPJ Digit Med. 2021;4(1):45. 33692479 10.1038/s41746-021-00418-3 PMC7946921

