# Supplementary material for: Lactate in Anaphylaxis: 100 Years On
Source: Sports Med. 2025 Jul 24;55(9):2091–110. doi: 10.1007/s40279-025-02273-8 (PMC12476325; doi:10.1007/s40279-025-02273-8)
Supplement: Supplementary file 1 — Supplementary file1 (PDF 478 KB) [file 40279_2025_2273_MOESM1_ESM.pdf]

Journal: Sports Medicine
Elena Borzova ORCID: https://orcid.org/0000-0003-1587-9137
Bernhard F. Gibbs ORCID: https://orcid.org/0000-0002-0537-228X

Title: **Lactate in anaphylaxis: 100 years on**

[1]Borzova E., [2]Gibbs B.F.

[1] Dermatology Division, Niigata University Graduate School of Medical and Dental Sciences, Niigata, Japan

[2] School of Psychology & Life Sciences, Canterbury Christ Church University, Canterbury, United Kingdom

Corresponding authors: Associate Professor Elena Borzova, Ph.D, Division of Dermatology, Niigata University Graduate School of Medical and Dental Sciences, 1-757 Asahimachi-dori, Chuo-ku, Niigata 951-8510, Japan. Email: elena-borzova@med.niigata-u.ac.jp, eborzova@gmail.com

Professor Bernhard F. Gibbs, Ph.D, School of Psychology & Life Sciences, North Holmes Road, Canterbury, Kent, CT1 1QU, UK. Email: Bernhard.gibbs@canterbury.ac.uk

Supplementary materials

Box S1. Criteria for hyperglycolytic states in cancer[82] and possibly other conditions

1. Increased glucose uptake and GLUT transporter expression
2. Upregulation of glycolytic enzymes
3. Decreased mitochondrial respiration
4. Increased lactate production, accumulation and release
5. Upregulation of MCT expression

Table S1 │Lactate production and signalling in mast cells

Reference	Year Ref. №	Mast cell (MC) source	Result
Hosoda & Glick	1965[51]	Mouse neoplastic MCs	Observed constitutive MC lactate dehydrogenase (LDH) activity and lactate formation
Diamant & Glick	1967[52]	Rat peritoneal MCs<br>Mouse neoplastic MCs	High levels of LDH activity in neoplastic MCs
Diamant & Peterson	1971[53]	Rat peritoneal MCs	Lactate accumulation and histamine release after stimulation with ATP. Involvement of glycolysis (Embden-Meyerhof pathway) in ATP-induced histamine release.
Chakravarty & Sorensen	1974[54]	Rat peritoneal MCs	Association with MC lactate production and histamine release following either IgE-dependent or compound 48/80-induced stimulation
Johansen	1979[55]	Rat peritoneal MCs	IgE-dependent histamine release is associated with decreased ATP levels. The use of glycolytic and respiratory inhibitors suggests that degranulation involves oxidative phosphorylation. Lactate levels did not, however, correlate with reduced cellular ATP levels.
Johansen	1980[46]	Rat peritoneal MCs	Compound 48/80-induced histamine release is associated with decreased ATP levels. Glycolytic and respiratory inhibitors inhibit lactate production in c48/80-stimulated MCs.
Johansen	1980[47]	Rat peritoneal MCs	Demonstrated that calcium ionophore A23187-induced MC degranulation is highly ATP-dependent and similar as previously reported for other IgE-dependent and -independent triggers. Lactate production increased during degranulation.

Fewtrell *et al.*	1981[56]	Rat basophilic leukaemia cells	LDH release from cells stimulated with high concentrations of either anti-IgE or A23187. Low pH reduced degranulation (serotonin release).
Johansen	1983[49]	Rat peritoneal MCs	Compound 48/80-induced histamine release is associated with significant production of lactate. Enhanced ATP-utilization even after degranulation.
Yoshizaki & Arizono	1991[48]	Rat peritoneal MCs	Glycolysis is activated after compound 48/80-induced MC histamine release and lactate production
Yoshizaki *et al.*	1993[50]	Rat peritoneal MCs	Lactate production and extracellular release from MCs is associated with both IgE-dependent and -independent histamine release.
Abebayehu *et al.*	2016[57]	Mouse bone marrow-derived MCs<br>Human skin MCs	Inhibition of IL-33-induced MC cytokine production by lactic acid. MCs express MCT-1, inhibitors of which block the suppressing effects of lactic acid. However, lactate had no effects, suggesting that pH plays a role for the inhibitory effects observed.
Abebayehu *et al.*	2019[58]	Mouse peritoneal MCs<br>Human skin MCs	Inhibition of IgE-mediated inflammatory cytokine production and degranulation due to lactic acid. Effects were dependent on MCT-1 and pH (no inhibitory effects with lactate). Lactic acid abrogates hypothermia in an *in vivo* passive systemic anaphylaxis mouse model.
Caslin et al.	2019[59]	Mouse bone marrow derived MCs<br>Mouse peritoneal MCs	Lactic acid inhibits TLR-mediated MC cytokine release, glycolysis, and pro-inflammatory cytokine release in a septic shock mouse model. Effects were dependent on MCT-1 and pH but

			high concentrations of lactate (>20mM) were also inhibitory.
Mendoza *et al.*	2021[60]	Mouse bone marrow-derived mast cells	In metabolomic studies, elevated lactate concentrations were reported in IgE-dependent MC activations but not in controls or following the stimulation with AgNP or CMD48/80.
Syed *et al.*	2021[61]	Mouse peritoneal MCs<br>Human skin MCs<br>LAD2 human MC line	Lactic acid inhibits MRGPRX2-mediated MC degranulation and chemokine/cytokine production. Lactic acid reduces compound 48/80-induced systemic anaphylaxis *in vivo*. The effects were largely due to the acidic properties of lactic acid. Lactate moderately reduced degranulation but not chemokine production.

Table S2 | Critical appraisal of lactate as a biomarker in anaphylaxis

Author	Anaphylaxis	Study design	Number of patients	Lactate detection	Lactate release	Clinical Evidence	Setting	Strengths	Limitations	Interpretation	Further validation
Hanashiro PK & Weil MH (1967)[134]	Drug-induced anaphylaxis	CR	2	Lactate to pyruvate ratio by modified enzymatic technique	Increased lactate/pyruvate ratio in two patients<br>Concentration of lactic acid and lactic pyruvate ratio in arterial blood was initially increased in both patients.<br>In the first patient lactic acid concentration was increased to 5.2 millimols/L<br>In the second patient, the ratio of lactate-tio-pyruvate was 3 at over 24 hours, with subsequent decline	Two case reports with initially increased lactic acid, followed by a gradual decrease	Shock Unit at the Los Angeles County General Hospital	Serial lactate measurements	Both patients received epinephrine before metabolic studies	A potential prognostic biomarker for a recovery from anaphylactic shock	Lactate monitoring in patients with anaphylaxis until the recovery
Tse KS et al (1980)[121]	Exercise-induced urticaria/angioedema	CR	1	Not reported	Post-challenge plasma lactate levels were 13 mmole/L	A parallel release of both lactate and histamine during an exercise challenge	Treadmill challenge setting (3.5 min, 4mph, with 12° grade]	Exercise challenge setting<br>Parallel histamine measurements	Limited clinical evidence	A potential early diagnostic biomarker for exercise-induced urticaria and angioedema	Prospective measurements with lactate wearables in athletes with or without exercise-induced anaphylaxis (EIA)<br>Real-time lactate and histamine measurements during exercise challenges in EIA patients and healthy subjects

Table S2│ Critical appraisal of lactate as a biomarker in anaphylaxis (*continued*)

Author	Anaphylaxis	Study design	Number of patients	Lactate detection	Lactate release	Clinical evidence	Setting	Strengths	Limitations	Interpretation	Further validation
Guerci P et al (2020)[23]	Fatal and near-fatal anaphylaxis	MC, R	339 patients,* including 17 patients who died of anaphylaxis	Not reported	Lactate > 6.9mM at ICU admission predicted death with a sensitivity of 70%, specificity of 87.5%, and positive predictive value of 80%	In multivariate analysis, only lactate concentration at ICU admission was a predictor of ICU mortality after Grade IV anaphylaxis (odds ratio: 1.47 [1.15-1.88], p=0.002)	23 ICUs in France within SFAR research network	A large national multi-center study of 339 anaphylactic patients<br>Multivariate modeling	Epinephrine-induced increase on serum lactate cannot be excluded in 277 patients, who were treated with epinephrine before ICU admission	A potential prognostic biomarker for fatal anaphylaxis	Lactate measurements in patients with anaphylaxis at ED or by ambulance teams
Perales-Chorda C et al (2021)[39]	Acute (<2 hours) and recovery phases (2-4 hours later) and a basal state (2-3 months after anaphylaxis) in patients with moderate and severe anaphylaxis	SC, P	18<br>(9 patients with moderate anaphylaxis and 8 patients with severe anaphylaxis, 1 patient with mild anaphylaxis)	Ultra-performance liquid chromatography coupled with mass spectrometry (UPLC-MS) and proton nuclear magnetic resonance spectrometry (^{1}H-NMR)	Individual lactate levels were not reported	32.62% of patients with moderate anaphylaxis had increase in serum lactate levels in acute phase compared to recovery phase (p=0.016).<br>In 2-3 months after an anaphylactic episode, there were significantly higher serum lactate levels in patients with severe anaphylaxis (35.17% higher) than in moderate anaphylaxis (p=0.029)	Outpatient clinics and Emergency departments and other services at Hospital La Fe (Valencia, Spain)	Metabolomic profiling<br>Prospective study design<br>Parallel tryptase measurements	Single center experience<br>Eight patients received epinephrine before metabolomic studies	A potential severity biomarker in anaphylaxis patients	Multi-center perspective studies in anaphylaxis patients with different severity

Table S2 | Critical appraisal of lactate as a biomarker in anaphylaxis (*continued*)

Author	Anaphylaxis	Study design	Number of patients	Lactate detection	Lactate release	Clinical evidence	Setting	Strengths	Limitations	Interpretation	Further validation
Boehm T et al (2021)[131]	Acute phase of a severe mast cell activation event (anaphylaxis) in a patient with indolent systemic mastocytosis	CR	1	UHPLC coupled with an Orbitrap Q Extractive mass spectrometry<br>Liquid chromatography mass spectrometry (LC-MS)	Lactate increase to 7mM at the peak of symptoms	Highly increased histamine and lactate concentrations at the peak of clinical symptoms	ED<br>ICU<br>at the Vienna General Hospital	Metabolomic profiling<br>Parallel histamine measurements	Limited clinical evidence	A potential biomarker of MC-related events in mastocytosis patients	Lactate measurements using wearables in mastocytosis patients with anaphylactic events
Oh HS et al (2024)[135]	Epinephrine-induced lactic acidosis in a patient with anaphylaxis, who received three doses of epinephrine	CR	1	Not reported	Lactate levels reached a peak at 13.5 mmol/L	Three case reports of anaphylactic patients with multiple epinephrine injections	ICU<br>At Samsung Medical Center, Seoul	Strong evidence in other contexts<br>Clinical relevance given 7.7% anaphylaxis patients receive multiple epinephrine injections	Lactate clearance in anaphylaxis patients, following multiple epinephrine injections, is unknown	A diagnostic biomarker of epinephrine-induced lactic acidosis	Lactate monitoring in patients with anaphylaxis who were received multiple doses of epinephrine

Abbreviations: CR, case report; MC, multi-center study; SC, single center study; P, prospective; R-retrospective, EIA, exercise-induced anaphylaxis; ED, emergency department; SFAR, Société Français d'Anesthésie-Réanimation.
*Including 62 patients without epinephrine injections prior to ICU admission

TABLE S3 | Lactate dehydrogenase in chronic inflammatory skin diseases

Disease	Study design	Patients	Lactate dehydrogenase			Relation to clinical indices	References
			Concentrations	Isozymes	Samples		
Atopic dermatitis	Longitudinal	58 children with atopic dermatitis	Increased LDH levels (n=13)	High LDH4 and LDH5 isozymes	Serum	Correlation with the severity of cutaneous symptoms (AD score, 0-15) (r=0.679) Decline in serum LDH levels following symptomatic improvement (6-12 months)	Morishima Y et al (2010)
		3 pediatric AD patients	Increased LDH activity in epidermis in the affected skin compared to healthy subjects	ND	Skin	AD score reported in three patients	
Atopic dermatitis	Interventional (dupilumab)	43 patients	Median baseline LDH 235 (129-340) U/L (n=27)	ND	Serum	There was a positive correlation between EASI score and serum LDH (p=0.026). Mean reductions in EASI score and LDH at 3-month follow-up were significantly correlated (p=0.003)	Olesen CM et al (2019)
Atopic dermatitis	Longitudinal Interventional (dupilumab) 3 pooled RCTs (DBPC): SOLO 1& 2, CHRONOS	1376 AD patients	Baseline LDH levels (median, 231.0-247.0) Increased LDH levels in 25.2-27% of AD patients in SOLO1&2 and 50.5-57.3% of AD patients in CHRONOS	ND	Serum	Baseline serum LDH correlates with AD disease activity and severity LDH was decreased during dupilumab studies in all three RCTs	Wollenberg A et al (2020)
Atopic dermatitis	Longitudinal Interventional (dupilumab)	54 adult patients with moderate to severe AD	Baseline LDH 269.0±93.2 U/L	ND	Serum	Baseline serum LDH levels were negatively correlated with the percentage reduction in EASI score at 3,6 and 12 months after initiating dupilumab (r=-0.59913; p=0.0003) Higher baseline LDH was associated with poor effectiveness of dupilumab	Kato A et al (2020)

						at 3 and 6 months	
Atopic dermatitis	Nationwide B-PAD RCT (dupilumab)	110 patients with moderate-to-severe AD	Baseline LDH (mean, SD): 264.8 (73.4) U/L	ND	Serum	Serum LDH showed the highest AUC for both POEM and pruritus-NRS	Nakahara T et al (2024)

Abbreviations: LDH, lactate dehydrogenase; B-PAD, Biomarkers to Predict Clinical Improvement of AD in Patients Treated with Dupilumab study; RCT, randomized controlled study; POEM, Patient-oriented Eczema Measure; AUC, area under the curve; ND, not done.

Additional reference list for Table S3:

1. Nakahara T, Onozuka D, Nunomura S, et al. The ability of biomarkers to assess the severity of atopic dermatitis. *J Allergy Clin Immunol Glob*. 2024;3(1):100175.
2. Morishima Y, Kawashima H, Takekuma K, Hoshika A. Changes in serum lactate dehydrogenase activity in children with atopic dermatitis. *Pediatr Int.* 2010;52(2):171-174.
3. Wollenberg A, Beck LA, Blauvelt A, Laboratory safety of dupilumab in moderate-to-severe atopic dermatitis: results from three phase III trials (LIBERTY AD SOLO 1, LIBERTY AD SOLO 2, LIBERTY AD CHRONOS). *Br J Dermatol* 2020;182(5):1120-1135.
4. Kato A, Kamata M, Ito M, et al. Higher baseline serum lactate dehydrogenase level is associated with poor effectiveness of dupilumab in the long term in patients with atopic dermatitis. *J Dermatol.* 2020;47(9):1013-1019.
5. Olesen CM, Holm JG, Nørreslet LB, Serup JV, Thomsen SF, Agner T. Treatment of atopic dermatitis with dupilumab: experience from a tertiary referral centre. *J Eur Acad Dermatol Venereol.* 2019;33(8):1562-1568.
